# Brain structure and function in *Homo naledi*

**DOI:** 10.1007/s00429-026-03129-1

**Published:** 2026-06-15

**Authors:** Zachary Cofran, Shawn Hurst, John Hawks

**Affiliations:** 1 Anthropology Department, Vassar College, Poughkeepsie, New York USA; 2 Department of Biology, University of Indianapolis, Indianapolis, Indiana USA; 3 Department of Anthropology, University of Wisconsin-Madison, Madison, Wisconsin USA

**Keywords:** Endocast, Geometric morphometrics, Cognition, Life history, Cerebellum

## Abstract

Endocasts are casts of the internal surface of skull bones created by the brain and surrounding tissues. These fossil phantoms provide the most direct evidence of the brains of extinct organisms, and are one of the many puzzle pieces that help paleoneurologists reconstruct brain evolution. The recent discovery of a small-brained, recently-extinct human species, *Homo naledi*, creates a timely opportunity to review what endocasts can and cannot tell us about ancient brains. We first review published evidence about the brain and behavior of *H. naledi*, including the suggestion that the species may have practiced mortuary behaviors over 230,000 years ago. We next use geometric morphometric methods to reconstruct and the most complete *H. naledi* endocast and compare it to those of modern humans and Pleistocene hominins. Our results corroborate previous evidence that the brain of *H. naledi* presents a unique combination of ancestral and modern human-like characteristics, specifically displaying a derived frontal lobe while retaining ancestral sizes, morphology, and cerebro-cerebellar proportions. Finally, we discuss these results in the context of recent paleoanthropological data, advances in neuroimaging, and theoretical frameworks linking brain morphology, structure, and function.

**Supplementary Information:**

The online version contains supplementary material available at 10.1007/s00429-026-03129-1.

## Introduction and background

A central question uniting disciplines across the natural and social sciences is the extent to which the human brain sets us apart from other animals. Paleoneurology, a subfield that infers brain structure and function based on fossils, brings critical information to this question because it examines the most direct evidence about the brains of extinct organisms: endocasts, or the casts created by the brain and surrounding tissues on the inside of the skull. The extinct human species *Homo naledi* (Berger et al. 2015), one of the latest additions to the human family tree, is an interesting case study in human paleoneurology because of its rich fossil record and unique paleontological context. Here, we present a new virtual reconstruction of the brain endocast from the most complete *H. naledi* cranium (“LES1”; Hawks et al. 2017), which we situate in the context of other evidence for human brain and behavioral evolution more broadly.

### *Homo naledi*: bones, brains, and behavior

The South African “Cradle of Humankind” is a rich system of karstic caves sprinkled across northern South Africa. Several cave sites bear geological deposits likely dating to between 1 and 3 million years ago (Pickering et al. 2019) or even 3.7 million years ago (Granger et al. 2015, 2022), providing crucial insights into early hominin evolution and diversity including the origins of our genus *Homo*. At most of these sites, hominins are but a fraction of the total fossil assemblages, and at many sites at least two hominin taxa are represented (Beaudet 2023; Braga and Moggi-Cecchi 2025; Brain 1970; Herries et al. 2020; Keyser et al. 2000). The 2013 discovery in Rising Star Cave of a trove of skeletal fragments, nearly all belonging to a single, previously unknown hominin species, was therefore quite unexpected (Berger et al. 2015). Over a decade of research has now shown that the taphonomic context of *H. naledi* in Rising Star Cave is unusual compared to other Cradle sites.

Most relevant to this study, taphonomic evidence suggests that *H. naledi* deliberately disposed of their dead in the Dinaledi Chamber, Lesedi Chamber, and other areas within Rising Star Cave (Berger et al. 2017, 2025; Brophy et al. 2021; Dirks et al. 2015; Hawks et al. 2017). These subterranean chambers are located in the ‘dark zone’ (i.e., receiving no natural light) 30 m below the Earth’s surface, and could only ever be accessed from today’s cave entrances located over 80 m away not including the twists and turns in between (Dirks et al. 2017). Navigating these tenebrous, tortuous tunnels may have required control of a light source (Randolph-Quinney 2015), i.e., fire. More recent archaeological work in the Dinaledi chamber further suggests individuals may have been *intentionally interred* in small pits (Berger et al. 2025). Radiometric and paleomagnetic dating of flowstones in the Dinaledi Chamber reveal that the *H. naledi* fossils were deposited between 241 and 335 kya (Dirks et al. 2017; Robbins et al. 2021). The proposal of intentional burial has been controversial, therefore, in part because this would constitute the earliest documented instance of such behavior (Martinón-Torres et al. 2023; Pettitt 2022; Val 2016). Nevertheless, geological and taphonomic evidence suggests that *H. naledi* individuals deliberately, and probably repeatedly, entered the cave’s deep recesses to dispose of their dead.

**Fig. 1:**
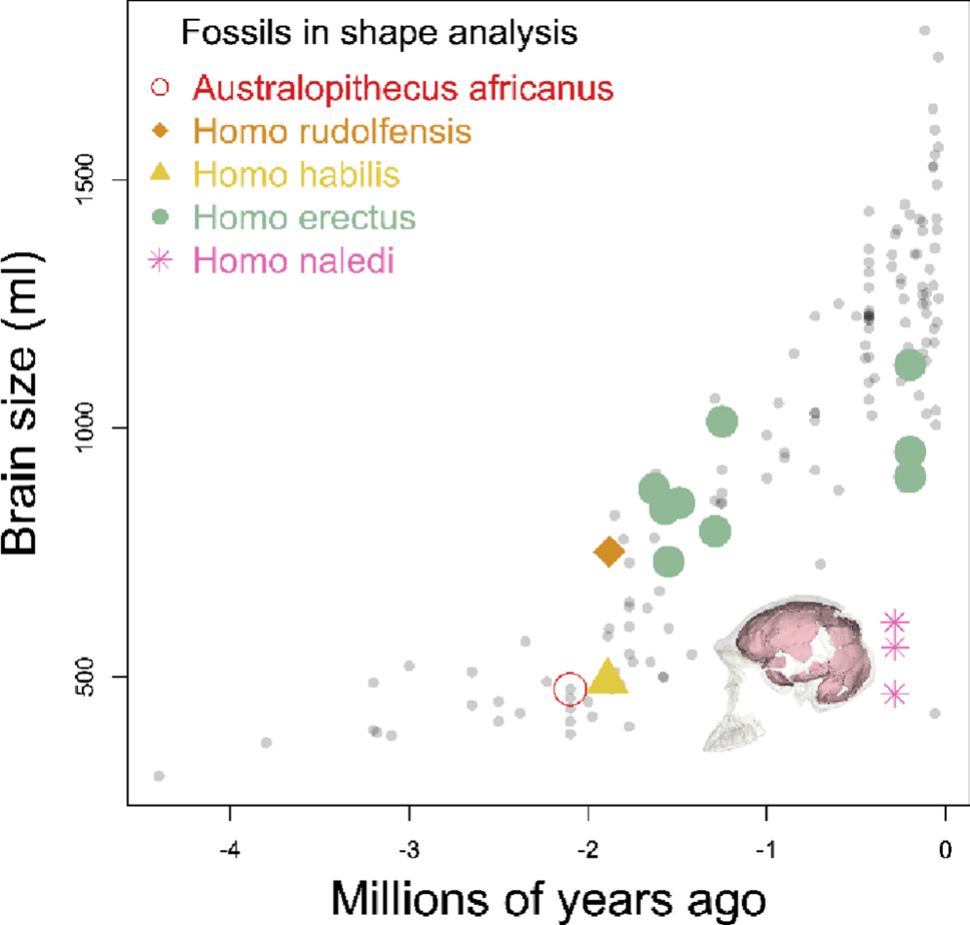
Hominin brain size evolution. Brain size (ECV, ml) for modern humans and fossil hominins (black circles), with *Homo naledi* and other fossil specimens included in this study indicated by unique colors and symbols. Data are modified from DeSilva et al. (2021)

**Fig. 2:**
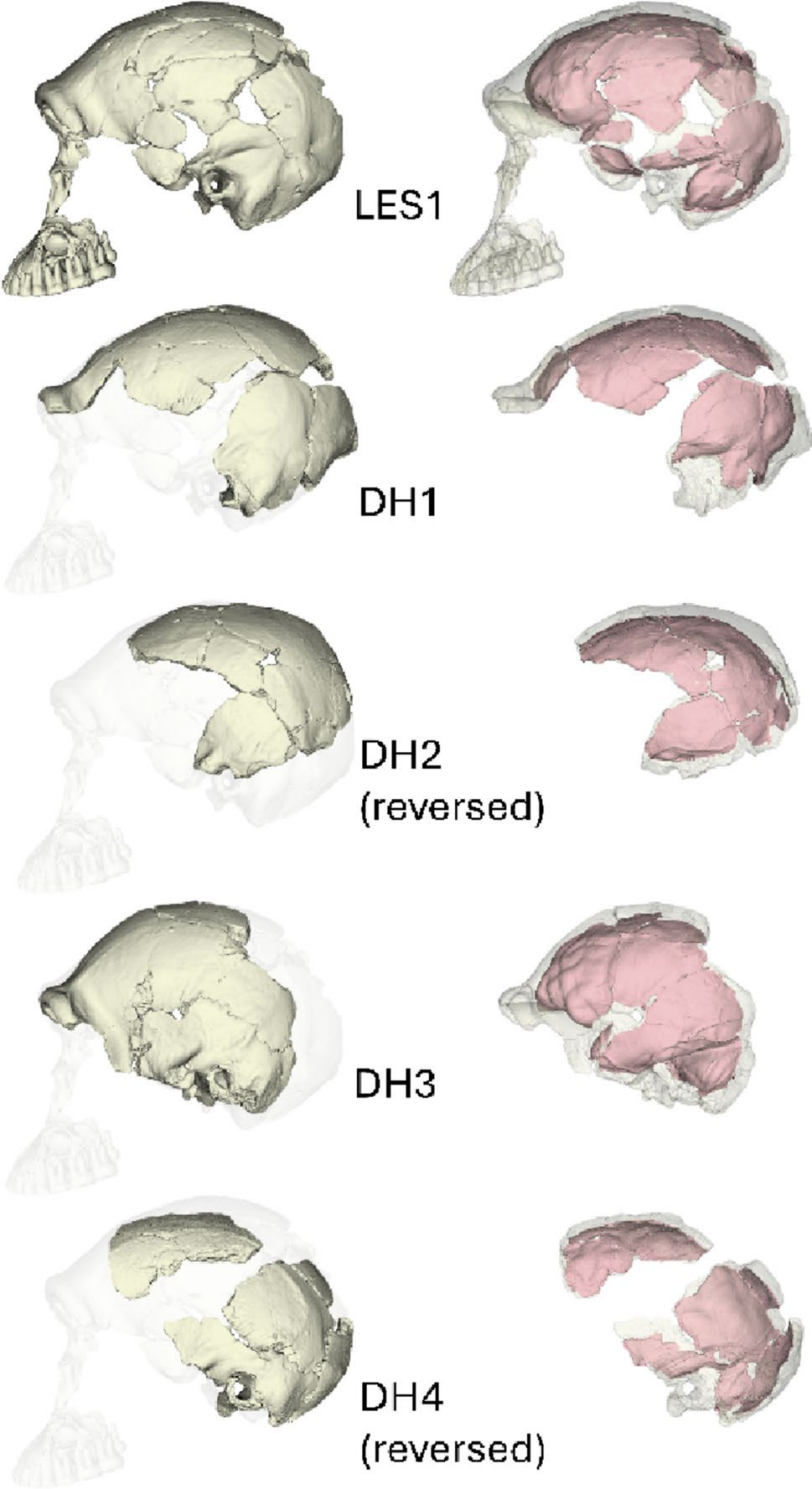
Partial crania (left) and endocasts (right) of *Homo naledi*. Each fossil is visually scaled to roughly the same size and positioned at the same antero-posterior anatomical position as LES1. Note that DH2 and DH4 preserve the right side of the cranium and are reversed here

The hypothesis that *H. naledi* practiced modern human-like mortuary behaviors has also been controversial because its fossil record retains many characteristics ancestral for the genus *Homo*, including a small brain size (Fig. 1). In primates including humans, the volume of the endocranial cavity (ECV, measured in milliliters, ml) is virtually identical to brain mass (measured in grams), making it a reliable proxy for brain size of extinct hominins (Isler et al. 2008). Four adult partial crania of *H. naledi* were initially recovered from the Dinaledi Chamber (Fig. 2), from which two composite crania were virtually reconstructed with computer-based methods, yielding estimated ECVs of 465 and 560 ml (Berger et al. 2015; Garvin et al. 2017). These estimates were later corroborated by nearly identical values (460 and 555 ml, respectively) based on physical reconstructions of these two composite endocasts (Holloway et al. 2018). Subsequent to these discoveries in the Dinaledi Chamber, the partial skull of another adult *H. naledi* individual, “LES1,” was recovered from the Lesedi Chamber of the cave (Hawks et al. 2017). The neurocranial vault of LES1 is much more complete than the Dinaledi crania, including a nearly complete frontal bone with much of the left orbital surface, nearly all of the left and and much of the right parietal bones, and fragmentary left temporal and occipital bones that directly refit (de Ruiter et al. 2019). A preliminary virtual reconstruction of the LES1 endocast yielded an ECV of 610 ml (Hawks et al. 2017).

Thus, *H. naledi* brain size estimates span 465–610 ml, which is within the range of australopiths that date to between 1.4 and 3.0 million years ago (Gunz et al. 2020; Holloway et al. 2004; Neubauer et al. 2012). These *H. naledi* brain size estimates overlap with only the smallest ECVs for *Homo* fossils that date to earlier than 1.5 million years ago (Benazzi et al. 2014; Lordkipanidze et al. 2013; Semaw et al. 2020), though even the lowest *H. naledi* estimate is larger than that of *Homo floresiensis* from the Late Pleistocene (Brown et al. 2004; Kubo et al. 2013). As we will discuss later, small brain size may have implications for cognition and life history in *H. naledi* (e.g., Deaner et al. 2007; Isler and van Schaik 2012).

**Fig. 3:**
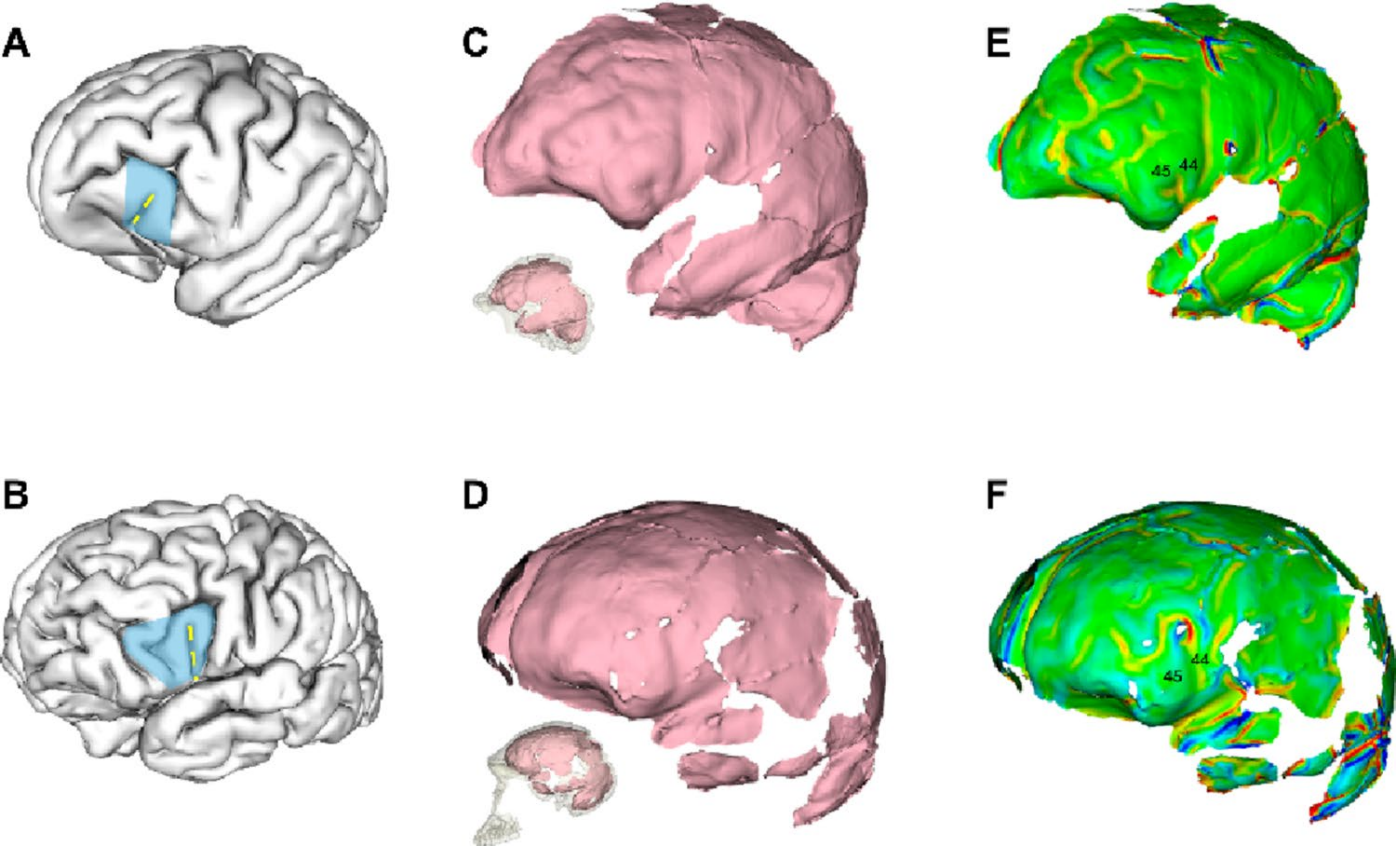
Frontal lobe morphology. Comparison of left frontal lobe morphology in anterior oblique view, highlighting Brodmann Areas 44–45 (blue in **A**, **B**). **A**: Chimpanzee cerebrum depicting the ancestral morphology. **B**: Human cerebrum depicting the derived morphology. In **A** and **B**, the dashed line indicates the sulcus typically separating area 44 rostrally from area 45 caudally. **C**, **D**: DH3 and LES1 endocasts, with inset showing the left lateral view. **E**, **F**: Color-coded curvature maps of DH3 and LES1, along with the presumed locations of BA44-45. Images are not to scale

In addition to size, endocast shape and surface impressions from cortical features can provide additional windows into the brains of extinct hominins (Dart 1925; Falk 2014; Holloway et al. 2004; Labra et al. 2024; Neubauer 2014; Zollikofer and Ponce de León, 2013). One of the most distinct morphological differences between the brains of humans versus the other apes lies in the inferior frontal gyrus (IFG; Fig. 3). The IFG includes Brodmann’s cytoarchitectonic areas 44–45 (BA 44 and 45) and is active in spoken language (Amunts and Zilles 2015; Sherwood et al. 2008) as well as stone tool production (Stout and Chaminade 2012). In humans and the other great apes, the IFG is bordered caudally by the inferior precentral sulcus and dorsally by the inferior frontal sulcus (Amunts et al. 1999; Cunningham and Horsley 1892; Hurst et al. 2025; Mingazzini 1928; Schenker et al. 2010; Sherwood et al. 2003; A. E. Walker and Fulton 1936). In non-human apes, BA 44 and 45 are typically, but not always, separated by a fronto-orbital sulcus (Schenker et al. 2010; Sherwood et al. 2003). In humans, however, expansion of the prefrontal cortex has resulted in BA 44–45 moving to a relatively more ventral and caudal position, creating a unique sulcal configuration separating these areas (Fig. 3) (Falk 2014; Holloway et al. 2018; Hurst et al. 2025).

Recent studies have suggested that many Early *Homo* endocasts exhibit the ancestral IFG sulcal anatomy shared with chimpanzees and *Australopithecus* (Beaudet and de Jager 2023; Ponce de León et al. 2021). Because small-brained crania tend to have stronger endocranial impressions of the brain and surrounding tissues (Connolly, 1950), the small brain size of *H. naledi* is fortuitous. Intriguingly, several *H. naledi* endocasts reveal derived frontal lobe anatomy, including a highly convoluted frontal cortex (DH3 and DH4; Fig. 2) and an enlarged IFG with a BA 44–45 morphology that anticipates the human condition (DH3 and LES1; Fig. 3) (Holloway et al. 2018; Hurst et al. 2024). The contrasting small ECV and modern human-like frontal lobe anatomy of *H. naledi* endocasts illustrate the complicated relationship between brain size and structural organization, a question going back to the origins of African paleoanthropology itself (Dart 1925; Beaudet 2025).

To gain new insights into the brain of *H. naledi*, we investigate the endocast of LES1 (Fig. 2) and compare its shape with that of humans and other fossil hominins, using 3D landmark-based geometric morphometrics (GM). Despite the impressive endocranial evidence for *H. naledi*, several aspects of its morphology warrant clarification that can be addressed with GM methods (Adams and Slice 2004; Bookstein 1992; Gunz et al. 2009; Neubauer 2014; Weber 2015). The GM toolkit allows a fragmentary fossil to be virtually reconstructed based on different reference specimens, which provides insight uncertainty due to missing data (Neubauer et al. 2012). Our first objective is therefore to use GM to reconstruct the entire endocast shape of LES1, including the missing basicranium and posterior-medial region, using different fossil references.

In addition to measuring biologically homologous landmarks such as endocranial foramina, GM methods have a robust framework for capturing the size and shape of biological curves (such as the lesser wing of the sphenoid that separates the anterior and middle cranial fossae) and relatively featureless surfaces (such as the dorsal endocast) via sliding “semilandmarks” that can be considered geometrically homologous (Gunz and Mitteroecker 2013; Gunz et al. 2005). The GM approach allows an entire endocast shape to be captured by the configuration of 3D landmark and semilandmark coordinates, in contrast to more traditional 2D linear measurements that are divorced from their geometric relationships (Baab et al. 2012; Neubauer 2014). Our second objective is therefore to situate the virtually reconstructed LES1 endocast in the context of Pleistocene hominin endocranial shape variation. Because skull shape (Schroeder et al. 2017) and tooth morphology (Delezene et al. 2023; Irish et al. 2018; Irish and Grabowski 2021) of *H. naledi* are generally most similar to Early Pleistocene *Homo* fossils, we hypothesize that the LES1 endocast will also share this affinity. In addition, we test whether *H. naledi* and other hominins follow the same scaling relationship as modern humans between the size of the cerebral cortex and cerebellum, as the latter region is of growing interest in human cognitive evolution (Barton 2012; Gunz et al. 2010; Kochiyama et al. 2018).

## Materials and methods

### LES1 endocast

**Fig. 4:**
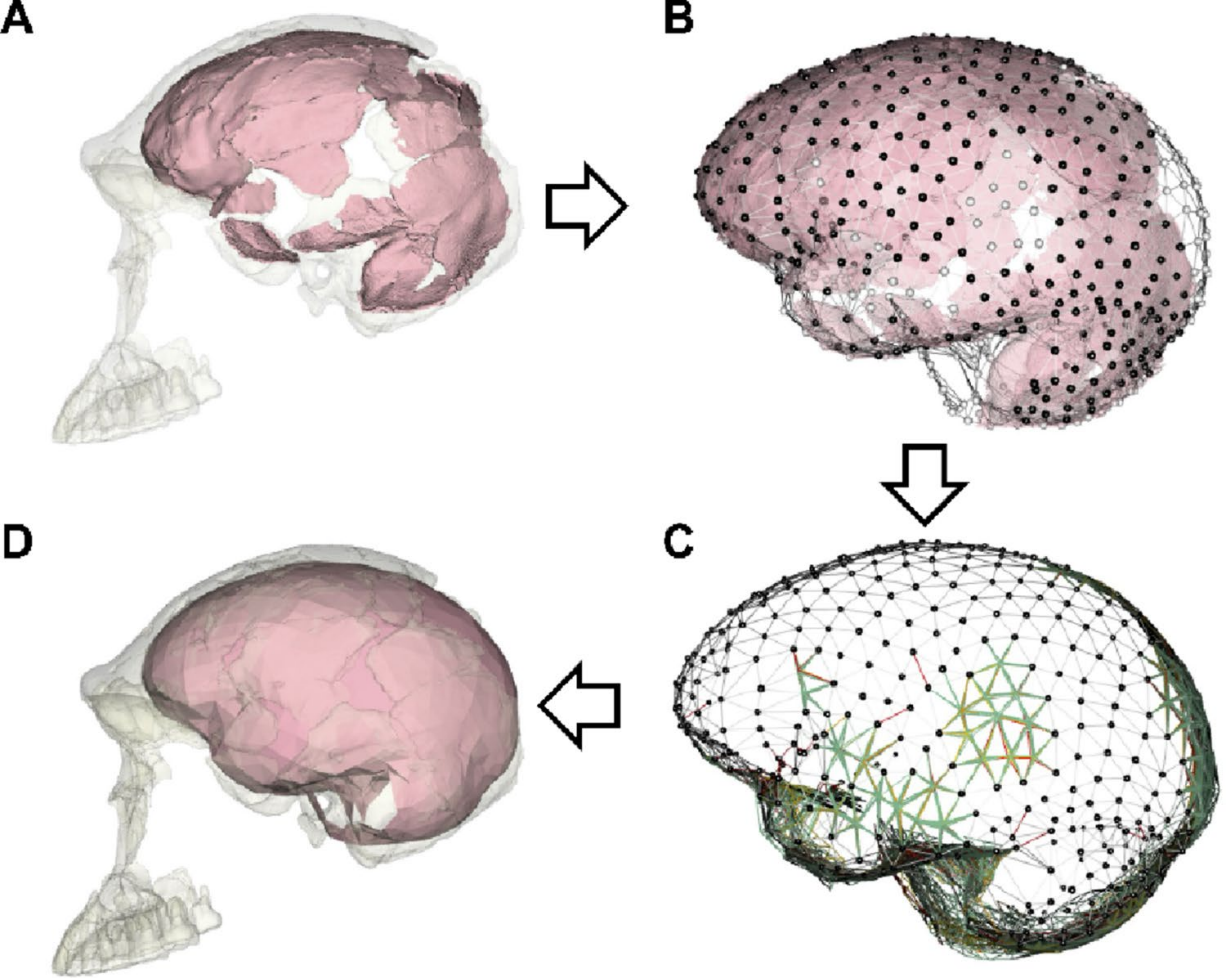
LES1 virtual reconstruction workflow. Graphical overview of the process of generating multiple endocast reconstructions and estimates of endocranial volume (see text for more details). Starting at the top left and moving counterclockwise: **A**: Original LES1 cranium in left lateral view, with the preserved endocranial surface highlighted in pink. **B**: The semi/landmark template (wireframe) overlain on the preserved, mirror-imaged LES1 endocast (pink transparent); preserved semi/landmarks are represented in black and missing data in white. **C**: The landmark template with all fossil-based reconstructions of missing data depicted as wireframes, color-coded to represent the group of the specific reference fossil (see other figures). **D**: Solid triangle mesh of the average LES1 virtual reconstruction occupying the endocranial cavity

We digitized the LES1 neurocranium (U.W. 102a-011) using an Artec Spider blue light surface scanner, and processed the resulting scans into a 3D mesh in Artec Studio 13 software (Fig. 4). Next, we deleted everything except for the endocranial surface of the mesh. We then duplicated the endocast mesh and mirror-imaged the copy, and then performed a best-fit alignment of the two surfaces in Artec Studio. This alignment is anchored by the well-preserved frontal and parietal surfaces on both sides.

### Landmark template

We used 3D landmark-based GM to capture the shape and estimate the positions of landmarks from regions not preserved in the mirror-imaged LES1 endocast (Gunz et al. 2009). We created and applied a landmark template to the LES1 endocast in Viewbox software (Halazonetis 2013), based on the average landmark configuration of 80 modern human adults from a previously published dataset (Neubauer et al. 2018a, b). The landmark template includes 29 fixed anatomical landmarks, 110 sliding semilandmarks distributed across two sagittal and four bilateral curves, and 796 sliding semilandmarks across the remaining endocast surface (Neubauer and Gunz 2018). The 796 surface semilandmarks are distributed symmetrically between the left and right sides.

We used an established protocol to fit the landmark template to the endocast in spite of missing data using Viewbox software (Bastir et al. 2019). Twenty-five of the 29 fixed landmarks from the template were not preserved, except for endobregma and the left and (mirrored) right transverse-sigmoid sinus junctions; the frontal crest was broken just superior to where the foramen caecum would have been located, and so this foramen landmark was manually estimated and positioned in Viewbox. Several of the important curves were at least partially preserved: the rostral half of the midsagittal curve, the lateral halves of the bilateral sphenoid curves, and most of the two bilateral curves for the superior margin of the transverse sinus and inferior margin of the transverse and sigmoid sinuses. We applied the landmark template (Fig. 4B), iteratively estimating the positions of missing landmarks by manually adjusting and automatically sliding landmarks and semilandmarks to minimize the bending energy of the thin plate spline interpolation between the LES1 configuration and reference template (Gunz and Mitteroecker 2013).

### Comparative sample

We compared the LES1 endocast to a previously published dataset of modern humans and fossil hominins using the same landmark template just described (Neubauer et al. 2018a, b). This sample includes 80 adult recent humans, one *H. habilis* (KNM-ER 1813), one *H. rudolfensis* (KNM-ER 1470), five African *H. erectus* (KNM-ER 3733, KNM-ER 3883, KNM-ER 42700, KNM-WT 15000, and OH 9), and five Indonesian *H. erectus* (Sangiran 2, Mojokerto, Sambungmacan 3, Ngawi, and Ngandong 14). This sample includes three non-adults (Mojokerto, KNM-ER 42700, and KNM-WT 15000), which are appropriate to include here as their smaller size (i.e., Mojokerto and KNM-ER 42700) is closer to that of LES1. This dataset includes two reconstructions of Mojokerto and seven of KNM-ER 42700, whose closeness to reality is unknowable. For our analysis, we arbitrarily selected the Mojokerto reconstruction where only missing data were estimated, and the “R3” reconstruction of KNM-ER 42,700 (further details of these reconstructions can be found in Neubauer et al. 2018a, b). We add to this sample the *Australopithecus africanus* specimen Sts 5, generated from a 3D surface mesh (Weber and Bookstein 2011) using the “endomaker” function of the *Arothron* package (Profico et al. 2020) in *R* version 4.1.2 (R Core Team, 2021). The comparative sample therefore includes 80 human adults and 13 fossil hominins, covering a range of brain sizes and shapes.

### Multiple reconstructions

With the landmark template applied to the LES1 endocast, we generated multiple landmark reconstructions based on the human average and each of the fossil individuals in the reference dataset (Fig. 4C). All landmark data were first imported into R software version 4.1.2 (R Core Team, 2021), and manipulated and analyzed using the packages *Morpho* (Schlager 2017), *geomorph* (Adams et al. 2025), *abind* (Plate and Heiberger 2024), and *Arothron* (Profico et al. 2020). We symmetrized the landmarks for LES1 and Sts 5 using the “symmetrize” function in *Morpho*. Symmetrization was necessary and appropriate because the LES1 reconstruction relied on mirror-imaging to reconstruct missing surfaces, and because the comparative dataset was previously symmetrized. We next identified the 356 landmarks not represented by the LES1 endocast and declared these as missing (“NA” in *R*). We then used the function “fixLMtps” from the *Morpho* package to estimate the positions of the missing landmarks using as references each of the other 13 hominin fossils, the average of all fossil hominins, and the modern human average, for a total of 15 reconstructions.

*Shape analysis*.

To explore shape affinities of the LES1 endocast, we first performed generalized Procrustes analysis of the full sample using the “gpagen” function in *geomorph.* This procedure centers, scales, and rotates the landmark configurations, superimposing them in a way that minimizes the average Procrustes shape distance (PD) between each individual and the sample average. PD is the standard measurement of difference or ‘distance’ between two landmark configurations (Baab et al. 2012).

We used these Procrustes coordinates to explore endocast shape variation. We first compared the pairwise PD among all of the LES1 reconstructions to the pairwise PDs in the rest of the sample, in order to assess the extent of missing data uncertainty and reliability of the LES1 reconstruction. We then examined shape in the sample in three ways, each using only the LES1 reconstruction in which missing data were estimated from the average of the fossil dataset. First, we compared its PD from all other fossils to the intragroup variation within both the human and *H. erectus* samples. These samples provide an empirical range of within-species variation to contextualize the shape similarities and differences between LES1 and the other fossils. Second, we performed hierarchical cluster analysis of the human and fossil sample based on the Procrustes distance matrix. We excluded the two immature *H. erectus* (Mojokerto and KNM-ER 42700) from the cluster analysis, as these were previously shown to have different endocranial shapes than the adolescent KNM-WT 15000 and adult *H. erectus* (Neubauer et al. 2018a, b). We used the unweighted pair group method with arithmetic mean (UPGMA) for clustering using the “hclust” function in the base statistics package of *R* (R Core Team, 2021). We performed cluster analysis both including all 80 humans and including only the human average, but here we present only results of the latter for ease of viewing since the topological positions of each group were roughly the same either way. Finally, we visualized the shape differences between LES1 and five endocast morphotypes: the modern human average, the *H. erectus* (*sensu lato*) average, *A. africanus*, *H. habilis*, and *H. rudolfensis*. Note that the last three are based on single fossil specimens rather than sample averages. To visualize how LES1 compares with the fossil sample, we superimposed triangulated surface meshes of LES1 and each morphotype, and color-coded the difference between homologous landmarks in each pairwise comparison using the “meshDist” function in the *Morpho* package (Schlager 2017).

Finally, we examined the relative sizes of the cerebral and cerebellar portions of the endocasts within our sample. The posterior cranial fossa houses the cerebellum and brain stem, and is osteologically demarcated anteriorly by the the petrous pyramids of the temporal bone and posteriorly by the transverse sinus of the occipital bone (Kubo et al. 2014). We used the landmarks covering the posterior cranial fossa as a proxy for the cerebellum, and the remaining landmarks (covering the endocranial vault and both anterior and middle cranial fossae) as a proxy for the cerebral cortex. Cerebrum and cerebellum size was calculated as the centroid size of the respective landmarks (Mitteroecker et al. 2013). We then plotted log_10_-transformed cerebellum versus cerebral centroid sizes and calculated the least-squares regression based on the human sample only, to assess whether fossil hominins follow the same pattern of scaling.

## Results

**Fig. 5:**
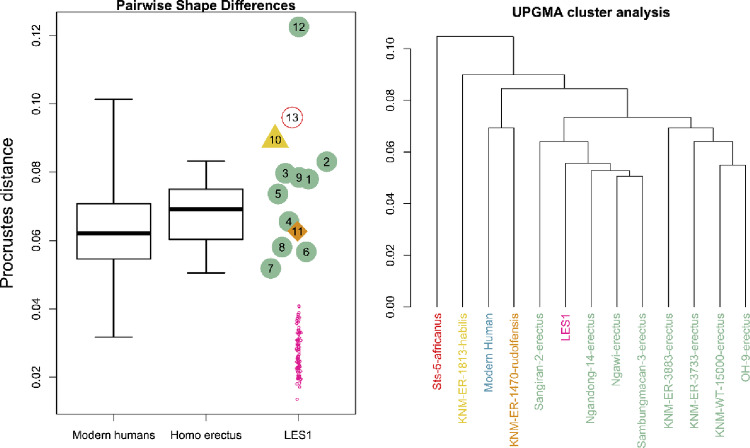
LES1 endocast shape affinities. Left: boxplot of pairwise Procrustes distances (PD) within specific samples: all 80 humans, all 8 adult *Homo erectus*, and LES1 average and each of the other 13 fossil hominins color-coded by taxon; pink dots indicate PDs between all LES1 reconstructions. The human and *Homo erectus* boxes and whiskers indicate adult intraspecific variation against which to consider the PDs between LES1 and each of the fossils. Right: Hierarchical cluster analysis of the adult sample and the LES1 average. Note that this cluster analysis includes the individual closest to the modern human average and not the full human sample. In the left panel numbers refer to fossil specimens: KNM-ER 42700 (1), KNM-ER 3733 (2), KNM-ER 3883 (3), KNM-WT 15000 (4), OH 9 (5), Ngandong 14 (6), Ngawi (7), Sambungmacan 3 (8), Sangiran 2 (9), KNM-ER 1813 (10), KNM-ER 1470 (11), Mojokerto (12), Sts 5 (13)

We used GM methods to produce multiple virtual reconstructions of the LES1 endocast, using 13 fossils and the human average as references. We compared pairwise shape differences within the sample quantified as the Procrustes distance (PD, Fig. 5). Procrustes distance (PD) between all LES1 reconstructions range from 0.0136 to 0.0409 (mean and median = 0.0274). These values are lower than all intraspecific PDs except 24/2500 (0.9%) of the human sample, lower than all PDs within the *H. erectus* sample, and far lower than the PDs between the LES1 average and all other fossils in the sample. Thus, error due to missing data is quite low for the LES1 endocast, meaning the virtual reconstruction likely provides an accurate estimate of its original size and shape.

Although *H. erectus* has higher intraspecific variation on average than humans, the range of pairwise PD is lower in *H. erectus*. LES1 is most similar to Ngawi (*H. erectus*), with a PD lower than the 50% interquartile range of either human or *H. erectus* intraspecific PD distributions. Pairwise PD between LES1 and all other *H. erectus* as well as *H. rudolfensis* specimen KNM-ER 1470 are all within the *H. erectus* intraspecific PD distribution. LES1 is most distinct from the fossils with the smallest endocranial volumes: KNM-ER 1813 (*H. habilis*), Sts 5, and the Mojokerto infant *H. erectus*.

**Fig. 6:**
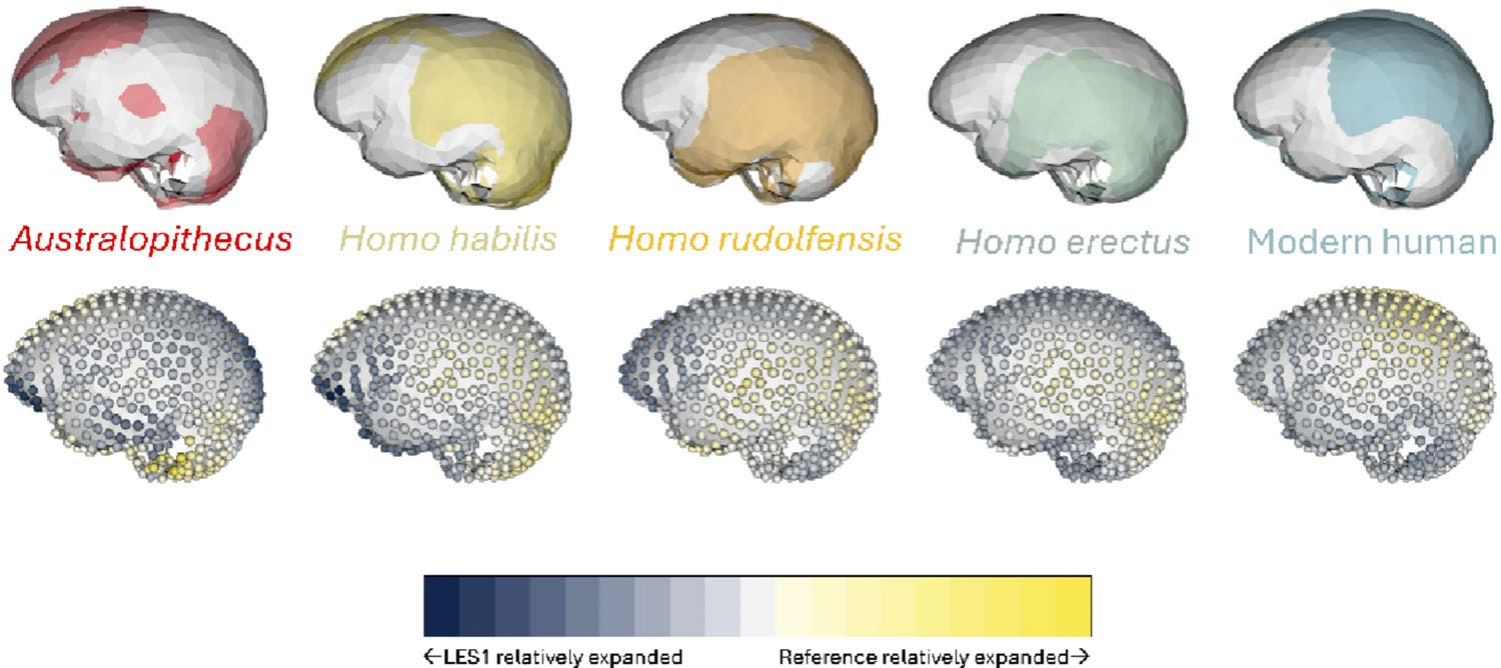
Comparison of endocranial morphotypes. Triangle meshes of the LES1 endocast compared with each of the five morphotypes defined in this study, from left to right: Sts 5 (*A. africanus*), KNM-ER 1813 (*H. habilis*), KNM-ER 1470 (*H. rudolfensis*), *H. erectus* average, and modern human average. Top row: Procrustes superimposition of LES1 (white) with each morphotype in semitransparent shading corresponding to the colors in previous figures. Bottom row: The LES1 endocast semi/landmarks colored along a scale depicting the distance between LES1 (white triangle mesh) and each morphotype: darker blues correspond to outward expansion in LES1 while brighter yellows indicate outward expansion in the compared morphotype. Endocasts are scaled to the same centroid size, so color depth is relative and unitless

To further explore shape similarities among hominins we also performed an UPGMA hierarchical cluster analysis of the PD matrix for the adult-only sample (i.e., excluding Mojokerto and KNM-ER 42700). Figure 5 shows the dendrogram from the cluster analysis with humans represented only by their overall average; this dendrogram differs from one derived using the entire human sample principally in the relative position of KNM-ER 1470 (Fig. S1). In Fig. 5, KNM-ER 1470 clusters with the modern human average due to its globular shape. However, carrying out the analysis with the full human sample, humans form their own cluster separate from one containing all fossil *Homo*, and KNM-ER 1470 forms a cluster with Indonesian *H. erectus* and LES1 (Fig. S1). In both cluster analyses, the following patterns emerge. First, Sts 5 forms its own branch separate from the rest of the sample and KNM-ER 1813 is on its own branch separate from all other fossil *Homo*, reflecting the distinctive shapes of each endocast (Fig. 6). Second, African and Indonesian *H. erectus* form their own distinct clusters (cf., Neubauer et al. 2018a, b). This difference is due to a relatively expanded frontal (i.e., inferiorly), relatively expanded parieto-occipital region, and relatively smaller posterior cranial fossa in the Indonesian sample (Fig. S2). Finally, LES1 falls within the Indonesian cluster, with the geologically older Sangiran 2 just outside of a subcluster including LES1 and the later Javanese fossils.

Thus, the LES1 endocast shape is most similar to that of *H. erectus* among the comparative sample, yet it is also distinct from each of the hominin morphotypes (Fig. S2). We compared the Procrustes shape coordinates of LES1 and each morphotype, depicted as both triangle meshes and color-coded distances between corresponding landmarks (Fig. 6). LES1 differs from the other morphotypes in its relatively expanded rostral and lateral frontal region, including in the vicinity of the IFG (Hurst et al. 2024). Because the original LES1 fossil is well preserved across this entire surface, this shape difference is not simply an artifact of the reconstruction process. Possibly related to this lateral frontal expansion, the mediolateral breadth of the LES1 endocast is fairly uniform from the rostral to the caudal ends, lacking the lateral ‘bulging’ of the parietotemporal region that characterizes other fossil *Homo* (Bruner and Holloway 2010).

**Fig. 7:**
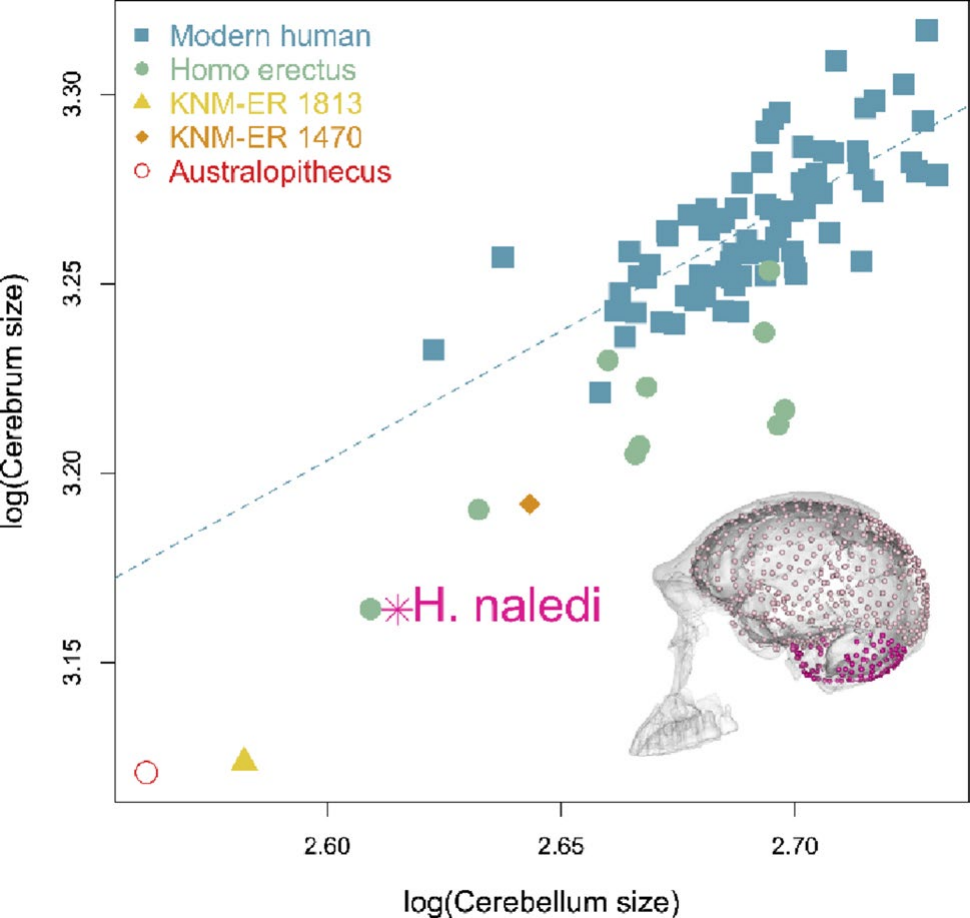
Cerebellum versus cerebral size. The two size variables are log_10_ centroid sizes of landmarks covering the cerebrum and cerebellum, respectively, as indicated on the LES1 endocast (inset). The regression line is fit to the adult human sample only, and colors/symbols correspond to samples as in other figures

In contrast to its derived frontal cortex morphology, the LES1 endocast appears like other fossil hominins in having a relatively small cerebrum compared to modern humans (Fig. 7). Within adult modern humans, cerebrum versus cerebellum size follows negative allometric scaling: log_10_(cerebrum) = 0.68 * log_10_(cerebellum) + 1.43 (R^2^ = 0.53, *P* = 9.1e^− 15^). Although we have not fit an allometric line to the fossil hominins, they appear to largely follow a similar scaling trend but with a lower y-intercept, i.e., smaller cerebra for a given cerebellum size. Four *H. erectus* specimens appear more like modern humans than the rest, however. Only Ngandong 14 falls within the human scatter. Ngawi, Sambungmacan 3, and OH9 similarly have enlarged cerebra compared to other *H. erectus*, falling just outside the margins of the human sample. The position of OH9 here is interesting as it is otherwise more like other African *H. erectus* in overall endocast shape (Fig. 5). Similarly, although the overall endocast shape of LES1 is most like the larger, later *H. erectus* from Indonesia, it nevertheless has a relatively smaller cerebrum like other fossil hominins.

In sum, our virtual reconstructions and analyses of the LES1 endocast reject our initial hypothesis that endocranial shape of *H. naledi* would be most similar to that of Early *Homo* (i.e., KNM-ER 1470 and KNM-ER 1813). Rather, LES1 endocast shared greater affinities with Indonesian *H. erectus*, whose brain sizes were 30–80% larger (Neubauer et al. 2018a, b). Nevertheless, LES1 is distinct in combining ancestral brain size and cerebrum-cerebellum proportions, with a relatively expanded rostral and lateral frontal surface. We discuss interpretations, implications, and limitations of these results below.

## Discussion

### Encephalization

The relatively complete skull and partial skeleton of the LES1 *H. naledi* provide a unique opportunity to review the relationship between body size, brain size, and brain structure in the human fossil record. The evolutionary significance of brain size has typically been examined in the context of ‘encephalization.’ Appreciating that brain size scales negatively allometrically with body size across species, Jerison (1955, 1973) developed the “encephalization quotient” (EQ) to quantify the extent to which a species may have a larger or smaller brain than expected for its body mass: a species’ EQ is the ratio of its observed brain size to that expected given its body size. Comparing EQ across species, it is assumed that there is a baseline amount of brain matter that must be dedicated to maintaining a body of a given size, and so the rationale behind EQ is that any brain mass beyond this allometric expectation could be given over to other cognitive functions. Though rarely explicated, these functions relate to receiving, processing, and acting upon different types, amounts, and novelty of information (Jerison 1973; Roth and Dicke 2005). Although there are different statistical approaches to measure encephalization (Grabowski et al. 2016; Isler et al. 2008), a practically universal result from decades of research is that hominins from australopiths to modern humans have larger brains than expected for primates of our body size (Aiello and Wheeler 1995; Alba 2010; Grabowski et al. 2016; Kappelman 1996; Mchenry, 1975, 1976; McHenry and Coffing 2000; Smaers et al. 2021).

Data from *H. naledi* help illustrate the challenges for estimating EQ in fossil taxa. Brain size is fairly straight-forward to estimate since endocranial volume almost perfectly approximates brain mass in primates (Isler et al. 2008). Body mass, however, is more difficult to estimate for extinct organisms (Grabowski et al. 2015; Ruff and Wood 2023). Load-bearing joints of the skeleton are typically used to estimate body mass for mechanical, empirical, and practical reasons. Because bipedal locomotion is a defining feature of the hominin clade, dimensions from throughout the lower limb are typically used to estimate body mass using regression equations developed from modern humans of known or estimated body mass (Ruff et al. 2018). The femoral head diameter of LES1 (C. S. Walker et al. 2019) predicts a body mass of 42.7 kg, using the ‘Worldwide’ equation developed by Ruff et al. (2018). However, because australopiths and some early *Homo* fossils, including *H. naledi* (Marchi et al. 2017), had relatively small femoral heads, Ruff et al. (2018) provided a further correction for estimating “non-*Homo*” specimens; this correction predicts 47.8 kg for LES1. Along these lines, *H. naledi* may have also habitually used their upper limbs for locomotion (Feuerriegel et al. 2019; Syeda et al. 2025), so it may also be reasonable to estimate body mass from the arm. Based on the humeral head diameter of LES1 (Feuerriegel et al. 2019; Ruff et al. 2020), body mass of LES1 is predicted to have been 40.5 kg. Thus, the LES1 hip and shoulder joints predict body masses between 40.5 and 47.8 kg with an average of 43.7 kg. Further factoring in the percent prediction errors of 7.2–7.8% reported for the prediction equations (Ruff and Wood 2023), a broader range of estimates for LES1 body mass is 37.3–51.2 kg. For comparison, this range is similar to average body masses of free-lived female and male chimpanzees, respectively (44–53 kg; Curry et al. 2023; 38–47 kg; Leigh 1994), and on the small end of modern human adult variation (R. J. Smith and Jungers 1997).

Plugging the brain size (610 ml) and body mass estimates based on the LES1 hip and shoulder joints into the phylogenetically-corrected best-fit equation of Grabowski and colleagues (2016) yields an EQ of 4.13 (femoral head), 3.86 (femoral head with correction), and 4.26 (humeral head); the broader range of body sizes incorporating prediction errors produces EQs from 3.70 to 4.48. This range overlaps or exceeds the high end of published *H. naledi* EQ estimates (3.4–4.1) based on the smaller Dinaledi brain sizes and larger postcranial sample, and is still lower than estimates for all other fossil *Homo* except *H*. *floresiensis* (Garvin et al. 2017; Grabowski et al. 2016). In spite of the variability in *H. naledi* EQ estimates, the relatively large sample size nevertheless provides a rough idea of its ‘true’ level of encephalization. EQ estimates for other hominin taxa are more dubious due to the combined effects of more limited sample sizes (e.g., *Ardipithecus ramidus*, *Australopithecus sediba*), different specimens used to estimate both brain and body size (i.e., *Au. sediba*, *Au. anamensis*), and uncertain taxonomic attribution of postcrania used to estimate body mass (i.e., *H. habilis*,* H. rudolfensis*,* Kenyanthropus platyops*). While these uncertainties and confounding factors hinder understanding the nuances of hominin encephalization, it is safe to conclude that absolute brain size of *H. naledi* was comparable to the earliest fossil *Homo*, but its relative brain size was likely more similar to australopiths, i.e., quite ancestral despite the chronological proximity of *H. naledi* to modern human origins.

#### Form and function

The cognitive implications of small absolute and relative brain size in *H. naledi* are also not straightforward. Some of this uncertainty stems from inherent and practical challenges of both defining and comparing cognitive performance. Researchers have crafted behavioral tests for comparing what could be thought of as problem-solving skills across a range of primate species (Deaner et al. 2006; Herrmann et al. 2007). On the one hand, some of these studies have shown that absolute and not relative brain size correlates best with performance (Deaner et al. 2007; MacLean et al. 2014). On the other hand, small-brained lemurs have performed as well as larger-brained haplorrhine primates on many physical and social cognitive tasks (Fichtel et al. 2020). An outstanding question is the extent to which such tests accurately capture the cognitive and behavioral capabilities of non-human animals (Schubiger et al. 2020). Nevertheless, there are clear limits to what we can infer about the cognition and behavior of extinct hominins on the basis of absolute and relative brain size.

Human cognition may arise from the emergent properties of an enlarged brain (Striedter 2006), and in many respects the human brain is a like scaled-up, general primate brain (Sherwood et al. 2012): e.g., numbers of neurons and other brain cells (Azevedo et al. 2009; Herculano-Houzel 2009; Herculano-Houzel et al. 2007); proportions of gray and white matter (Ardesch et al. 2022; Zhang and Sejnowski 2000); and folding of some areas of the neocortex (Herculano-Houzel et al. 2010). Morphologically, however, the hominin cortex deviates from general primate patterns. Human newborns and infants have brain sizes comparable to those of chimpanzee and gorilla adults (Cofran 2019; Cofran and DeSilva 2015), yet even at birth human endocranial shape is distinct from that of other apes at all ages (Neubauer et al. 2010; Scott et al. 2014). Moreover, characteristic human cerebral morphology, including an IFG operculating the insula, are present from before birth (Cohen-Sacher et al. 2006; Connolly, 1950; Cunningham and Horsley 1892; Garcia et al. 2018; Holloway et al. 2018 Supporting Information).

Similarly, adult *H. naledi* brain sizes are within the upper 75% of modern gorillas (Isler et al. 2008; McFarlin et al. 2013), yet *H. naledi* endocasts present a more convoluted frontal anatomy including a larger IFG with a modern human-like sulcus arrangement. In these respects, LES1 and other *H. naledi* appear more derived than similarly-sized *Australopithecus* and Early *Homo* (KNM-ER 3732: Beaudet and de Jager 2023; KNM-ER 1805: Falk 1983; Ponce de León et al. 2021). In addition, the overall brain shape of LES1, as reflected in its endocast, is not simply a “scaled-down” *H. erectus* brain at a smaller size (Neubauer et al. 2018a, b). Although we have not directly compared LES1 with the geologically older and slightly larger Early *Homo* endocast from Dmanisi, the latter appear to share the posterior parietal flattening and lateral bulging with *H. erectus*, and possibly an ancestral IFG morphology (Ponce de León et al. 2021). Hurst and colleagues (2024) also found that *H. naledi* endocasts DH3 and LES1 present an enlarged IFG, despite using a very different measurement protocol. This is especially intriguing since IFG size appears to increase proportionally with ECV from small-brained extant *Pan* through *Australopithecus* and *Homo* (Balzeau et al. 2014). The combination of a gorilla-sized brain with a derived frontal lobe size and anatomy in *H. naledi* supports long-standing arguments that hominin brain structure could be derived while retaining ancestral sizes (Dart 1925).

A major caveat to this discussion, however, is that endocranial form is influenced not only by the brain and surrounding tissues, but also by the brain’s spatial relationship to other functional matrices such as the orbits, nasopharynx, oral cavity, and spinal cord (Bruner 2014; Bruner and Holloway 2010; Moss and Young 1960; Pereira-Pedro et al. 2017; Zollikofer et al. 2017). Furthermore, the semilandmarks covering much of the endocranial surface that we used in our analyses are geometrically homologous and together describe endocranial form, but do not necessarily correspond with homologous points on the brain’s cortical surface (Gunz and Mitteroecker 2013). Thus, another interpretation of the relatively inflated lateral frontal region of the LES1 endocast (Fig. 6) is that it does not reflect an enlarged IFG, but rather is a byproduct of how the frontal lobe is arranged above the orbits.

The DAN5/P1 *H. erectus* cranium, dating to 1.5 million years ago, is an interesting point of comparison here (Semaw et al. 2020). Like LES1, the DAN5/P1 endocast presents a brain size of approximately 600 ml and shows less of a discrepancy between frontal and parietal widths compared to *H. erectus* (Bruner et al. 2023). A recent GM analysis of Middle Pleistocene hominin crania found that the LES1 midface was most similar to that of DAN5/P1, but the two were nevertheless distinct from one another in their overall cranial shape (Baab et al. 2025).This latter result suggests a different relationship between the face and braincase in LES1 and DAN5/P1, which obscures how their similar frontal widening may have been influenced by cranial architecture. Alternatively, it has previously been shown that much of the variation in endocranial proportions within the genus *Homo* can be at least partly explained by overall brain size (Bruner et al. 2015; Bruner and Holloway 2010). It is therefore possible that similarities in endocranial form between LES1 and DAN5 reflect their small overall size. These questions can be illuminated by future work directly examining both endocranial allometry (cf., Neubauer et al. 2018a, b) and the relationships between craniofacial and endocranial form (cf., Zollikofer et al. 2022) in comprehensive samples of fossil hominins.

Another way to uncover the relationship between endocranial shape and brain structure is to look within modern brains. Advances in neuroscience over the past three decades indicate that variation in gyral and sulcal morphology reflects structural differences arising from connections across the cortex, among other physical factors (Van Essen 1997; Essen 2020). During growth of the cerebral cortical sheet, folding occurs due to disproportionate growth relative to the underlying white matter connecting different regions of the cortex (Garcia et al. 2021; Holland et al. 2015; Solhtalab et al. 2025; Toro and Burnod 2005; Yin et al. 2025). In turn, cortical geometry and connectivity are associated with brain function (Baruzzi et al. 2023; Buckner and Krienen 2013; Pang et al. 2023; Schwartz et al. 2023; Stout and Hecht 2017). A fruitful approach to studying fossil endocasts may be to view cortical convolutions as representing specific patterns of connectivity across the cortex (Changeux et al. 2021), rather than as circumscribed areas with specific cognitive functions and associated behaviors (Hayden et al. 2025). The mechanical and developmental basis of different cortical folding patterns in humans, macaques, and ferrets is well enough understood that it can be simulated in physical gel models and virtual computational models (Tallinen et al. 2016; Yin et al. 2025). It may be possible, then, to explore how patterns of cortical connectivity and size expansion might result in morphologies of *H. naledi* and other fossil hominins. Thus, we may be able to work backward starting from cortical morphology represented by endocasts to reconstruct aspects of their internal structure (Yin et al. 2025), and thereby ultimately gain new insights about how hominin brains functioned.

Our results also encourage further investigation into the evolution of the hominin cerebellum. Historically assumed to relate mainly to basic motor functions, the cerebellum’s importance for language and other behaviors, as well as its communication with association areas of the cerebral cortex, is increasingly appreciated (Buckner and Krienen 2013; Rilling 2006; Strick et al. 2009; Tanabe et al. 2018). Indeed, despite its smaller volume and lesser historical attention, the cerebellum actually has far more neurons and surface area than the cerebral cortex (Herculano-Houzel 2009; Sereno et al. 2020). The cerebellum has expanded dramatically in size among hominoids compared to other primates (Barton and Venditti 2014; Rilling and Insel 1998; Weaver 2005), and the modern human cerebellum appears expanded relative to Neandertals (Cofran et al. 2021; Kochiyama et al. 2018; Kubo et al. 2014; Tanabe et al. 2018). Human brain ontogeny shortly after birth also differs from apes and even Neandertals due in part to disproportionate expansion of the cerebellum, as represented by the posterior cranial fossa (Gunz et al. 2010, 2012, 2019; Neubauer et al. 2010; Scott et al. 2014). Our allometric analysis indicates that modern humans have a larger cerebral cortex than expected for cerebellum size compared to the fossil sample. Our study did not include Neandertals, but given the patterns just described and the fact that Neandertal brain sizes were comparable to those of modern humans (VanSickle et al. 2020), we might expect that Neandertals would continue beyond the fossil hominin allometric trajectory ‘beneath’ humans in Fig. 7, or possibly plot above the lower end of the human scatter (cf., Bienvenu 2010). A more focused analysis with a larger fossil sample, especially of Middle Pleistocene hominins that are not attributed to *H. erectus* or Neandertals, could provide crucial details about the timing and behavioral correlates of cerebellar evolution in the human lineage.

Beyond making inferences about cognition and behavioral capacities from fossil impressions alone, we can also draw on paleontological and archaeological clues about the actual behaviors of *H. naledi* and other hominins (Wynn and Coolidge 2016). As noted earlier, geological evidence reveals that *H. naledi* may have intentionally placed bodies of conspecifics deep in the dark and tortuous Rising Star Cave system, repeatedly over several generations (Dirks et al. 2017). In addition, hand phalanges of *H. naledi* have cortical bone distribution, which adapts to forces experienced during life, indicating habitual lithic production and use (Syeda et al. 2025), although no clear lithic culture has been discovered in Rising Star Cave. Nevertheless, these behaviors imply the ability to imagine an outcome, plan for the materials and sequence of events necessary to achieve that outcome, and then successfully execute the sequence. The derived IFG of *H. naledi* is salient here because Brodmann’s areas 44–45 in the IFG are implicated in chimpanzee vocal production (Amiez et al. 2023) and full-blown human language (Friederici 2023); language areas and networks of the brain are also recruited in stone tool production (Stout and Chaminade 2012; Stout and Hecht 2017). Thus, the relatively enlarged orbitofrontal region and derived IFG anatomy of *H. naledi* could represent the neural substrates through which individuals engaged in ‘advanced’ behaviors requiring imagining, planning, and executing large action sequences. It is tempting to speculate further, e.g., that *H. naledi* may be at least partially responsible for the contemporaneous Middle Stone Age (Berger et al. 2017) often heralded as the potential origins of modern humans’ behavioral flexibility and capacity for culture (McBrearty and Brooks, 2000; Scerri and Will 2023). Similarly, if *H. naledi* did in fact intentionally dispose of the dead in a special location (“funerary caching”), this could indicate the evolution of memory and “cultural understanding of death” (Pettitt 2024, p. 516). Such speculation is probably premature, however, given how widespread both culture (Laland and Hoppitt 2003) and mortuary activity are throughout the animal kingdom (Pettitt 2018).

#### Life history

Finally, brain size has implications for life history strategy, or the adaptive allocation of energy through the life course (Burger et al. 2019; Charnov and Berrigan 1993). Brain tissue is notoriously energetically expensive (Aiello and Wheeler 1995; Holliday 1986). Isler and van Schaik (2012) have suggested apes with brain sizes over 700 g (or ml) would require more calories for dependent offspring to grow than could be supported with uniparental (i.e., maternal) support, as is characteristic of most primate species. All *H. naledi* ECVs are below this brain size “gray ceiling,” beyond which cooperative breeding would theoretically be necessary to maintain viable population sizes. While this implies that *H. naledi* would not have required cooperative breeding to ensure offspring survival, it does not preclude it.

Similarly, high rates of postnatal brain size growth in modern humans are a critical part of our ‘slow’ life history strategy (Gurven and Walker 2005; Kaplan et al. 2000). These high brain growth rates during infancy comes at the cost of slow and prolonged juvenile body size growth (Kuzawa et al. 2014). High rates of brain size growth were probably absent in *Australopithecus* (Alemseged et al. 2006; Cofran 2019; Gunz et al. 2020), but evolved alongside larger adult brain size in Early Pleistocene *H. erectus* (Cofran and DeSilva 2015; Leigh 2006; Zollikofer and Ponce de León 2013). With its smaller adult brain sizes, *H. naledi* may also be expected to have had slower rates of brain size growth. Admittedly indirect evidence from dental development suggests that both small-brained australopiths and larger-brained Early Pleistocene *H. erectus* experienced more rapid bodily growth (Dean and Smith 2009; B. H. Smith 1986). Unexpectedly, dental development in *H. naledi* is more similar to humans in ways that have been associated with our slow life history strategy (Cofran and Walker 2017; Guatelli-Steinberg et al. 2018; Mahoney et al. 2024). If dental maturation can indeed serve as a proxy for body maturation (but see T. M. Smith 2013; Zollikofer et al. 2024), *H. naledi* may then present a unique life history strategy in which small brain size and slow maturation served to reduce caloric costs of brain growth and metabolism.

## Conclusions

*Homo naledi* is one of the many recently discovered branches in the hominin family tree, and this branch appears to be especially leafy. The large fossil record for this species provides a unique view of its biological variation, including that of the brain. Here, we used the well preserved LES1 endocast to situate *H. naledi* in the contexts of both hominin evolution and of modern paleoneurology. As with any great fossil discovery, the combination of ancestral and derived features in the *Homo naledi* brain raises new questions and points to new avenues of investigation. Advances in neuroimaging and computational simulations are promising approaches for reconstructing the structure and function of ancient brains.

## Electronic Supplementary Material

Below is the link to the electronic supplementary material.

Supplementary Material 1

## Data Availability

We used landmark data from Neubauer, Simon, 2018, “KNM-ER 42700: endocranial data”, accessible at https://doi.org/10.17617/3.18. Landmark data and R code novel to this study are available in the Zenodo repository, accessible at https://zenodo.org/records/20136569.

## References

[CR2] Adams D, Collyer M, Kaliontzopoulou A, Baken E (2025) Geomorph: geometric morphometric analyses of 2D and 3D landmark data. Retrieved from https://cran.r-project.org/web/packages/geomorph/index.html

[CR1] Adams DC, Rohlf F, James,and, Slice DE (2004) Geometric morphometrics: Ten years of progress following the ‘revolution’. Italian J Zool 71(1):5–16. 10.1080/11250000409356545

[CR3] Aiello LC, Wheeler P (1995) The Expensive-Tissue Hypothesis: The Brain and the Digestive System in Human and Primate Evolution. Curr Anthropol 36(2):199–221

[CR4] Alba DM (2010) Cognitive inferences in fossil apes (Primates, Hominoidea). J Anthropol Sci 88:11–48 20834049

[CR5] Alemseged Z, Spoor F, Kimbel WH, Bobe R, Geraads D, Reed D, Wynn JG (2006) A juvenile early hominin skeleton from Dikika, Ethiopia. Nature 443(7109):296–301. 10.1038/nature05047 16988704 10.1038/nature05047

[CR6] Amiez C, Verstraete C, Sallet J, Hadj-Bouziane F, Hamed B, Meguerditchian S, Hopkins A, W. D (2023) The relevance of the unique anatomy of the human prefrontal operculum to the emergence of speech. Commun Biology 6(1):1–12. 10.1038/s42003-023-05066-9 10.1038/s42003-023-05066-9 PMC10322890 37407769

[CR7] Amunts K, Schleicher A, Bürgel U, Mohlberg H, Uylings HBM, Zilles K (1999) Broca’s region revisited: Cytoarchitecture and intersubject variability. J Comp Neurol 412(2):319–341. 10.1002/(SICI)1096-9861)412:2%253C319::AID-CNE10%253E3.0.CO;2-7 10441759 10.1002/(sici)1096-9861(19990920)412:2<319::aid-cne10>3.0.co;2-7

[CR8] Amunts K, Zilles K (2015) Architectonic Mapping of the Human Brain beyond Brodmann. Neuron 88(6):1086–1107. 10.1016/j.neuron.2015.12.001 26687219 10.1016/j.neuron.2015.12.001

[CR9] Ardesch DJ, Scholtens LH, de Lange SC, Roumazeilles L, Khrapitchev AA, Preuss TM, van den Heuvel MP (2022) Scaling Principles of White Matter Connectivity in the Human and Nonhuman Primate Brain. Cereb Cortex 32(13):2831–2842. 10.1093/cercor/bhab384 34849623 10.1093/cercor/bhab384 PMC9247419

[CR10] Azevedo FAC, Carvalho LRB, Grinberg LT, Farfel JM, Ferretti REL, Leite REP, Herculano-Houzel S (2009) Equal numbers of neuronal and nonneuronal cells make the human brain an isometrically scaled-up primate brain. J Comp Neurol 513(5):532–541. 10.1002/cne.21974 19226510 10.1002/cne.21974

[CR12] Baab KL, Kaifu Y, Freidline SE, Rogers MJ, Semaw S (2025) New reconstruction of DAN5 cranium (Gona, Ethiopia) supports complex emergence of Homo erectus. Nat Commun 16(1):10878. 10.1038/s41467-025-66381-9 41402278 10.1038/s41467-025-66381-9 PMC12708782

[CR11] Baab KL, McNulty KP, Rohlf FJ (2012) The shape of human evolution: A geometric morphometrics perspective. Evolutionary Anthropology: Issues News Reviews 21(4):151–165. 10.1002/evan.21320 10.1002/evan.21320 22907868

[CR13] Balzeau A, Gilissen E, Holloway RL, Prima S, Grimaud-Hervé D (2014) Variations in size, shape and asymmetries of the third frontal convolution in hominids: Paleoneurological implications for hominin evolution and the origin of language. J Hum Evol 76:116–128. 10.1016/j.jhevol.2014.06.006 25042287 10.1016/j.jhevol.2014.06.006

[CR14] Barton RA (2012) Embodied cognitive evolution and the cerebellum. Philosophical Trans Royal Soc B: Biol Sci 367(1599):2097–2107. 10.1098/rstb.2012.0112 10.1098/rstb.2012.0112 PMC3385677 22734053

[CR15] Barton RA, Venditti C (2014) Rapid Evolution of the Cerebellum in Humans and Other Great Apes. Curr Biol 24(20):2440–2444. 10.1016/j.cub.2014.08.056 25283776 10.1016/j.cub.2014.08.056

[CR16] Baruzzi V, Lodi M, Sorrentino F, Storace M (2023) Bridging functional and anatomical neural connectivity through cluster synchronization. Sci Rep 13(1):22430. 10.1038/s41598-023-49746-2 38104227 10.1038/s41598-023-49746-2 PMC10725511

[CR17] Bastir M, García-Martínez D, Torres-Tamayo N, Palancar CA, Fernández-Pérez FJ, Riesco-López A, López-Gallo P (2019) Workflows in a Virtual Morphology Lab: 3D scanning, measuring, and printing. J Anthropol Sci. 10.4436/JASS.97003 31472012 10.4436/JASS.97003

[CR18] Beaudet A (2023) The *Australopithecus* assemblage from Sterkfontein Member 4 (South Africa) and the concept of variation in palaeontology. Evolutionary Anthropology: Issues News Reviews 32(3):154–168. 10.1002/evan.21972 10.1002/evan.21972 36632711

[CR19] Beaudet A, de Jager E (2023) Broca’s area, variation and taxic diversity in early *Homo* from Koobi Fora (Kenya). eLife 12:RP89054. 10.7554/eLife.89054 37721480 10.7554/eLife.89054 PMC10506792

[CR185] Beaudet A, de Jager E, Tawane M, Billings B (2025) Looking for the origins of the human brain: The role of South Africa in the history of palaeoneurology. South Afr J Sci 121(1/2)

[CR20] Benazzi S, Gruppioni G, Strait DS, Hublin J-J (2014) Technical Note: Virtual reconstruction of KNM-ER 1813 *Homo habilis* cranium. Am J Phys Anthropol 153(1):154–160. 10.1002/ajpa.22376 24318950 10.1002/ajpa.22376

[CR21] Berger LR, Hawks J, de Ruiter DJ, Churchill SE, Schmid P, Delezene LK, Zipfel B (2015) *Homo naledi*, a new species of the genus Homo from the Dinaledi Chamber, South Africa. eLife 4:e09560. 10.7554/eLife.09560 26354291 10.7554/eLife.09560 PMC4559886

[CR22] Berger LR, Hawks J, Dirks PH, Elliott M, Roberts EM (2017) *Homo naledi* and Pleistocene hominin evolution in subequatorial Africa. eLife 6:e24234. 10.7554/eLife.24234 28483041 10.7554/eLife.24234 PMC5423770

[CR23] Berger LR, Makhubela T, Molopyane K, Krüger A, Randolph-Quinney P, Elliott M, Hawks J (2025) Evidence for deliberate burial of the dead by Homo naledi. ELife 10.7554/eLife.89106.2 10.7554/eLife.89106 PMC12401548 40888484

[CR24] Bienvenu T (2010) *L*’Endocrâne de Sahelanthropus Tchadensis (Hominidae, Miocène supérieur du Tchad): Reconstitution 3D et morphologie: comparaison avec les hominoïdes actuels et fossiles (Ph.D., L’UNIVERSITE DE POITIERS). L’UNIVERSITE DE POITIERS. Retrieved from https://theses.fr/2010POIT2301

[CR25] Bookstein FL (1992) Morphometric Tools for Landmark Data: Geometry and Biology. Cambridge University Press, Cambridge. 10.1017/CBO9780511573064

[CR26] Braga J, Moggi-Cecchi J (2025) Infant craniofacial diversity in Early Pleistocene *Homo*. Nat Commun 16(1):4796. 10.1038/s41467-025-59734-x 40461481 10.1038/s41467-025-59734-x PMC12134133

[CR27] Brain CK (1970) New Finds at the Swartkrans Australopithecine Site. Nature 225(5238):1112–1119. 10.1038/2251112a0 5418243 10.1038/2251112a0

[CR28] Brophy JK, Elliott MC, Ruiter DJD, Bolter DR, Churchill SE, Walker CS, Berger LR (2021) Immature Hominin Craniodental Remains From a New Locality in the Rising Star Cave System, South Africa. PaleoAnthropology. 10.48738/2021.iss1.64

[CR29] Brown P, Sutikna T, Morwood MJ, Soejono RP, Jatmiko W, Saptomo E, Due A, R (2004) A new small-bodied hominin from the Late Pleistocene of Flores, Indonesia. Nature 431(7012):1055–1061. 10.1038/nature02999 15514638 10.1038/nature02999

[CR30] Bruner E (2014) Functional craniology, human evolution, and anatomical constraints in the neanderthal braincase. In: T. Akazawa, N. Ogihara, H. C Tanabe, & H. Terashima (Eds.), Dynamics of Learning in Neanderthals and Modern Humans Volume 2: Cognitive and Physical Perspectives. Springer Japan. pp 121–129 10.1007/978-4-431-54553-8_13

[CR31] Bruner E, Grimaud-Hervé D, Wu X, de la Cuétara JM, Holloway R (2015) A paleoneurological survey of Homo erectus endocranial metrics. Quaternary Int Groundw Cult Herit 368:80–87. 10.1016/j.quaint.2014.10.007

[CR32] Bruner E, Holloway R, Baab KL, Rogers MJ, Semaw S (2023) The endocast from Dana Aoule North (DAN5/P1): A 1.5 million year-old human braincase from Gona, Afar, Ethiopia. Am J Biol Anthropol 181(2):206–215. 10.1002/ajpa.24717 36810873 10.1002/ajpa.24717

[CR33] Bruner E, Holloway RL (2010) A bivariate approach to the widening of the frontal lobes in the genus Homo. J Hum Evol 58(2):138–146. 10.1016/j.jhevol.2009.10.005 20035967 10.1016/j.jhevol.2009.10.005

[CR34] Buckner RL, Krienen FM (2013) The evolution of distributed association networks in the human brain. Trends Cogn Sci 17(12):648–665. 10.1016/j.tics.2013.09.017 24210963 10.1016/j.tics.2013.09.017

[CR35] Burger JR, Hou C, Brown JH (2019) Toward a metabolic theory of life history. Proc Natl Acad Sci 116(52):26653–26661. 10.1073/pnas.1907702116 31822607 10.1073/pnas.1907702116 PMC6936346

[CR36] Changeux J-P, Goulas A, Hilgetag CC (2021) A Connectomic Hypothesis for the Hominization of the Brain. Cereb Cortex 31(5):2425–2449. 10.1093/cercor/bhaa365 33367521 10.1093/cercor/bhaa365 PMC8023825

[CR37] Charnov EL, Berrigan D (1993) Why do female primates have such long lifespans and so few babies? Or Life in the slow lane. Evolutionary Anthropology: Issues News Reviews 1(6):191–194. 10.1002/evan.1360010604

[CR38] Cofran Z (2019) Brain size growth in *Australopithecus*. J Hum Evol 130:72–82. 10.1016/j.jhevol.2019.02.006 31010545 10.1016/j.jhevol.2019.02.006

[CR39] Cofran Z, Boone M, Petticord M (2021) Virtually estimated endocranial volumes of the Krapina Neandertals. Am J Phys Anthropol 174(1):117–128. 10.1002/ajpa.24165 33111974 10.1002/ajpa.24165

[CR40] Cofran Z, DeSilva JM (2015) A neonatal perspective on *Homo erectus* brain growth. J Hum Evol 81:41–47. 10.1016/j.jhevol.2015.02.011 25771994 10.1016/j.jhevol.2015.02.011

[CR41] Cofran Z, Walker CS (2017) Dental development in *Homo naledi*. Biol Lett 13(8):20170339. 10.1098/rsbl.2017.0339 28855415 10.1098/rsbl.2017.0339 PMC5582112

[CR42] Cohen-Sacher B, Lerman-Sagie T, Lev D, Malinger G (2006) Sonographic developmental milestones of the fetal cerebral cortex: A longitudinal study. Ultrasound Obstet Gynecol 27(5):494–502. 10.1002/uog.2757 16619380 10.1002/uog.2757

[CR43] Connolly CJ (1950) External Morphology of the Primate Brain. Springfield, Ill.: C. C. Thomas. Retrieved from https://catalog.hathitrust.org/Record/001576611

[CR44] Cunningham DJ, Horsley V (1892) Contribution to the Surface Anatomy of the Cerebral Hemispheres. Academy House

[CR45] Curry BA, Drane AL, Atencia R, Feltrer Y, Howatson G, Calvi T, Shave R (2023) Body mass and growth rates in captive chimpanzees (Pan troglodytes) cared for in African wildlife sanctuaries, zoological institutions, and research facilities. Zoo Biol 42(1):98–106. 10.1002/zoo.21718 35815730 10.1002/zoo.21718 PMC10084351

[CR46] Dart RA (1925) *Australopithecus africanus* The Man-Ape of South Africa. Nature 115(2884):195–199. 10.1038/115195a0

[CR49] Deaner RO, Isler K, Burkart J, van Schaik C (2007) Overall Brain Size, and Not Encephalization Quotient, Best Predicts Cognitive Ability across Non-Human Primates. Brain Behav Evol 70(2):115–124. 10.1159/000102973 17510549 10.1159/000102973

[CR50] Deaner RO, van Schaik CP, Johnson V (2006) Do Some Taxa Have Better Domain-General Cognition than others? A Meta-Analysis of Nonhuman Primate Studies. Evolutionary Psychol 4:149–196

[CR48] Dean MC, Smith BH (2009) Growth and Development of the Nariokotome Youth, KNM-WT 15000. In F. E. Grine, J. G. Fleagle, & R. E. Leakey (Eds.), The First Humans – Origin and Early Evolution of the Genus Homo: Contributions from the Third Stony Brook Human Evolution Symposium and Workshop October 3 – October 7, 2006. Dordrecht: Springer Netherlands. pp 101–120 10.1007/978-1-4020-9980-9_10

[CR51] Delezene LK, Skinner MM, Bailey SE, Brophy JK, Elliott MC, Gurtov A, Berger LR (2023) Descriptive catalog of *Homo naledi* dental remains from the 2013 to 2015 excavations of the Dinaledi Chamber, site U.W. 101, within the Rising Star cave system, South Africa. J Hum Evol 180:103372. 10.1016/j.jhevol.2023.103372 37229947 10.1016/j.jhevol.2023.103372

[CR47] de Ruiter DJ, Laird MF, Elliott M, Schmid P, Brophy J, Hawks J, Berger LR (2019) *Homo naledi* cranial remains from the Lesedi chamber of the rising star cave system, South Africa. J Hum Evol 132:1–14. 10.1016/j.jhevol.2019.03.019 31203841 10.1016/j.jhevol.2019.03.019

[CR52] DeSilva JM, Traniello JFA, Claxton AG, Fannin LD (2021) When and why did human brains decrease in size? a new change-point analysis and insights from brain evolution in ants. Front Ecol Evol 9. 10.3389/fevo.2021.742639

[CR53] Dirks PH, Berger LR, Roberts EM, Kramers JD, Hawks J, Randolph-Quinney PS, Tucker S (2015) Geological and taphonomic context for the new hominin species *Homo naledi* from the Dinaledi Chamber, South Africa. eLife 4:e09561. 10.7554/eLife.09561 26354289 10.7554/eLife.09561 PMC4559842

[CR54] Dirks PH, Roberts EM, Hilbert-Wolf H, Kramers JD, Hawks J, Dosseto A, Berger LR (2017) The age of *Homo naledi* and associated sediments in the Rising Star Cave, South Africa. eLife 6:e24231. 10.7554/eLife.24231 28483040 10.7554/eLife.24231 PMC5423772

[CR55] Falk D (1983) Cerebral Cortices of East African Early Hominids. Science 221(4615):1072–1074 17736656 10.1126/science.221.4615.1072

[CR56] Falk D (2014) Interpreting sulci on hominin endocasts: Old hypotheses and new findings. Front Hum Neurosci 8. 10.3389/fnhum.2014.00134 10.3389/fnhum.2014.00134 PMC4013485 24822043

[CR57] Feuerriegel EM, Voisin J-L, Churchill SE, Haeusler M, Mathews S, Schmid P, Berger (2019) and L. R. Upper Limb Fossils of *Homo naledi* from the Lesedi Chamber, Rising Star System, South Africa. *PaleoAnthropology*, *2019*, 311–349

[CR58] Fichtel C, Dinter K, Kappeler PM (2020) The lemur baseline: How lemurs compare to monkeys and apes in the Primate Cognition Test Battery. PeerJ 8:e10025. 10.7717/peerj.10025 33024643 10.7717/peerj.10025 PMC7520086

[CR59] Friederici AD (2023) Evolutionary neuroanatomical expansion of Broca’s region serving a human-specific function. Trends Neurosci 46(10):786–796. 10.1016/j.tins.2023.07.004 37596132 10.1016/j.tins.2023.07.004

[CR60] Garcia KE, Robinson EC, Alexopoulos D, Dierker DL, Glasser MF, Coalson TS, Bayly PV (2018) Dynamic patterns of cortical expansion during folding of the preterm human brain. *Proceedings of the National Academy of Sciences*, *115*(12), 3156–3161. 10.1073/pnas.1715451115 10.1073/pnas.1715451115 PMC5866555 29507201

[CR61] Garcia KE, Wang X, Kroenke CD (2021) A model of tension-induced fiber growth predicts white matter organization during brain folding. Nat Commun 12(1):6681. 10.1038/s41467-021-26971-9 34795256 10.1038/s41467-021-26971-9 PMC8602459

[CR62] Garvin HM, Elliott MC, Delezene LK, Hawks J, Churchill SE, Berger LR, Holliday TW (2017) Body size, brain size, and sexual dimorphism in *Homo naledi* from the Dinaledi Chamber. J Hum Evol 111:119–138. 10.1016/j.jhevol.2017.06.010 28874266 10.1016/j.jhevol.2017.06.010

[CR63] Grabowski M, Hatala KG, Jungers WL, Richmond BG (2015) Body mass estimates of hominin fossils and the evolution of human body size. J Hum Evol 85:75–93. 10.1016/j.jhevol.2015.05.005 26094042 10.1016/j.jhevol.2015.05.005

[CR64] Grabowski M, Voje KL, Hansen TF (2016) Evolutionary modeling and correcting for observation error support a 3/5 brain-body allometry for primates. J Hum Evol 94:106–116. 10.1016/j.jhevol.2016.03.001 27178462 10.1016/j.jhevol.2016.03.001

[CR65] Granger DE, Gibbon RJ, Kuman K, Clarke RJ, Bruxelles L, Caffee MW (2015) New cosmogenic burial ages for Sterkfontein Member 2 *Australopithecus* and Member 5 Oldowan. Nature 522(7554):85–88. 10.1038/nature14268 25830884 10.1038/nature14268

[CR66] Granger DE, Stratford D, Bruxelles L, Gibbon RJ, Clarke RJ, Kuman K (2022) Cosmogenic nuclide dating of *Australopithecus* at Sterkfontein, South Africa. *Proceedings of the National Academy of Sciences*, *119*(27), e2123516119. 10.1073/pnas.2123516119 10.1073/pnas.2123516119 PMC9271183 35759668

[CR67] Guatelli-Steinberg D, O’Hara MC, Le Cabec A, Delezene LK, Reid DJ, Skinner MM, Berger LR (2018) Patterns of lateral enamel growth in *Homo naledi* as assessed through perikymata distribution and number. J Hum Evol 121:40–54. 10.1016/j.jhevol.2018.03.007 29709292 10.1016/j.jhevol.2018.03.007

[CR68] Gunz P, Mitteroecker P (2013) Semilandmarks: A method for quantifying curves and surfaces. Hystrix Italian J Mammalogy 24(1):103–109. 10.4404/hystrix-24.1-6292

[CR69] Gunz P, Mitteroecker P, Bookstein FL (2005) Semilandmarks in Three Dimensions. In D. E. Slice (Ed.), Modern Morphometrics in Physical Anthropology (pp. 73–98). Boston, MA: Springer US. 10.1007/0-387-27614-9_3

[CR70] Gunz P, Mitteroecker P, Neubauer S, Weber GW, Bookstein FL (2009) Principles for the virtual reconstruction of hominin crania. J Hum Evol 57(1):48–62. 10.1016/j.jhevol.2009.04.004 19482335 10.1016/j.jhevol.2009.04.004

[CR71] Gunz P, Neubauer S, Falk D, Tafforeau P, Le Cabec A, Smith TM, Alemseged Z (2020) *Australopithecus afarensis* endocasts suggest ape-like brain organization and prolonged brain growth. Sci Adv 6(14):eaaz4729. 10.1126/sciadv.aaz4729 32270044 10.1126/sciadv.aaz4729 PMC7112758

[CR72] Gunz P, Neubauer S, Golovanova L, Doronichev V, Maureille B, Hublin J-J (2012) A uniquely modern human pattern of endocranial development. Insights from a new cranial reconstruction of the Neandertal newborn from Mezmaiskaya. J Hum Evol 62(2):300–313. 10.1016/j.jhevol.2011.11.013 22221766 10.1016/j.jhevol.2011.11.013

[CR73] Gunz P, Neubauer S, Maureille B, Hublin J-J (2010) Brain development after birth differs between Neanderthals and modern humans. Curr Biol 20(21):R921–R922. 10.1016/j.cub.2010.10.018 21056830 10.1016/j.cub.2010.10.018

[CR74] Gunz P, Tilot AK, Wittfeld K, Teumer A, Shapland CY, van Erp TGM, Fisher SE (2019) Neandertal Introgression Sheds Light on Modern Human Endocranial Globularity. Curr Biol 29(1):120–127e5. 10.1016/j.cub.2018.10.065 30554901 10.1016/j.cub.2018.10.065 PMC6380688

[CR75] Gurven M, Walker R (2005) Energetic demand of multiple dependents and the evolution of slow human growth. Proc Royal Soc B: Biol Sci 273(1588):835–841. 10.1098/rspb.2005.3380 10.1098/rspb.2005.3380 PMC1560216 16618677

[CR76] Halazonetis D (2013) *Viewbox*. Kifissia, Greece: dHAL Software. Retrieved from https://www.dhal.com/viewbox.htm

[CR77] Hawks J, Elliott M, Schmid P, Churchill SE, de Ruiter DJ, Roberts EM, Berger LR (2017) New fossil remains of *Homo naledi* from the Lesedi Chamber, South Africa. eLife 6:e24232. 10.7554/eLife.24232 28483039 10.7554/eLife.24232 PMC5423776

[CR78] Hayden BY, Heilbronner SR, Yoo SBM (2025) Rethinking the centrality of brain areas in understanding functional organization. Nat Neurosci 1–12. 10.1038/s41593-025-02166-z 10.1038/s41593-025-02166-z PMC13032191 41436651

[CR79] Herculano-Houzel S (2009) The human brain in numbers: A linearly scaled-up primate brain. Front Hum Neurosci 3. 10.3389/neuro.09.031.2009 10.3389/neuro.09.031.2009 PMC2776484 19915731

[CR80] Herculano-Houzel S, Collins CE, Wong P, Kaas JH (2007) Cellular scaling rules for primate brains. *Proceedings of the National Academy of Sciences*, *104*(9), 3562–3567. 10.1073/pnas.0611396104 10.1073/pnas.0611396104 PMC1805542 17360682

[CR81] Herculano-Houzel S, Mota B, Wong P, Kaas JH (2010) Connectivity-driven white matter scaling and folding in primate cerebral cortex. *Proceedings of the National Academy of Sciences*, *107*(44), 19008–19013. 10.1073/pnas.1012590107 10.1073/pnas.1012590107 PMC2973896 20956290

[CR82] Herries AIR, Martin JM, Leece AB, Adams JW, Boschian G, Joannes-Boyau R, Menter C (2020) Contemporaneity of *Australopithecus*, *Paranthropus*, and early *Homo erectus* in South Africa. Science 368(6486):eaaw7293. 10.1126/science.aaw7293 32241925 10.1126/science.aaw7293

[CR83] Herrmann E, Call J, Hernàndez-Lloreda MV, Hare B, Tomasello M (2007) Humans Have Evolved Specialized Skills of Social Cognition: The Cultural Intelligence Hypothesis. Science 317(5843):1360–1366. 10.1126/science.1146282 17823346 10.1126/science.1146282

[CR84] Holland MA, Miller KE, Kuhl E (2015) Emerging Brain Morphologies from Axonal Elongation. Ann Biomed Eng 43(7):1640–1653. 10.1007/s10439-015-1312-9 25824370 10.1007/s10439-015-1312-9 PMC4497873

[CR85] Holliday MA (1986) Body Composition and Energy Needs during Growth. In: Falkner F, Tanner JM (eds) Postnatal Growth Neurobiology. Springer US, Boston, MA, pp 101–117. 10.1007/978-1-4899-0522-2_5

[CR86] Holloway RL, Broadfield D, Yuan M (2004) *The Human Fossil Record: Brain Endocasts—The Paleoneurological Evidence*. John Wiley & Sons, Ltd. 10.1002/0471663573.app1

[CR87] Holloway RL, Hurst SD, Garvin HM, Schoenemann PT, Vanti WB, Berger LR, Hawks J (2018) Endocast morphology of Homo naledi from the Dinaledi Chamber, South Africa. Proc Natl Acad Sci 115(22):5738–5743. 10.1073/pnas.1720842115 29760068 10.1073/pnas.1720842115 PMC5984505

[CR88] Hurst S, Holloway R, Garvin H, Bocko G, Garcia K, Cofran Z, Berger L (2025) A reanalysis of the Taung endocranial surface: Comparison with large samples of living hominids. J Hum Evol 200:103637. 10.1016/j.jhevol.2024.103637 39965466 10.1016/j.jhevol.2024.103637

[CR89] Hurst S, Holloway RL, Balzeau A, Garvin HM, Vanti WB, Berger LR, Hawks J (2024) The endocast morphology of LES1, *Homo naledi*. Am J Biol Anthropol 184(4):e24983. 10.1002/ajpa.24983 38864146 10.1002/ajpa.24983

[CR90] Irish JD, Bailey SE, Guatelli-Steinberg D, Delezene LK, Berger LR (2018) Ancient teeth, phenetic affinities, and African hominins: Another look at where *Homo naledi* fits in. J Hum Evol 122:108–123. 10.1016/j.jhevol.2018.05.007 29887210 10.1016/j.jhevol.2018.05.007

[CR91] Irish JD, Grabowski M (2021) Relative tooth size, Bayesian inference, and Homo naledi. Am J Phys Anthropol 176(2):262–282. 10.1002/ajpa.24353 34190335 10.1002/ajpa.24353

[CR152] Sereno MI, Diedrichsen I, Tachrount M, Testa-Silva G, d’Arceuil H, De Zeeuw C (2020) The human cerebellum has almost 80% of the surface area of the neocortex. Proc Natl Acad Sci 117(32):19538–19543. 10.1073/pnas.2002896117 32723827 10.1073/pnas.2002896117 PMC7431020

[CR92] Isler K, Christopher Kirk E, Miller JMA, Albrecht GA, Gelvin BR, Martin RD (2008) Endocranial volumes of primate species: Scaling analyses using a comprehensive and reliable data set. J Hum Evol 55(6):967–978. 10.1016/j.jhevol.2008.08.004 18817943 10.1016/j.jhevol.2008.08.004

[CR93] Isler K, van Schaik CP (2012) How Our Ancestors Broke through the Gray Ceiling: Comparative Evidence for Cooperative Breeding in Early Homo. Curr Anthropol 53(S6):S453–S465. 10.1086/667623

[CR94] Jerison HJ (1955) Brain to Body Ratios and the Evolution of Intelligence. Science 121(3144):447–449. 10.1126/science.121.3144.447 14358669 10.1126/science.121.3144.447

[CR95] Jerison HJ (1973) *Evolution of the Brain and Intelligence*. New York: Academic Press. Retrieved from http://p5070-www.sciencedirect.com/book/monograph/9780123852502/evolution-of-the-brain-and-intelligence

[CR96] Kaplan H, Hill K, Lancaster J, Hurtado AM (2000) A theory of human life history evolution: Diet, intelligence, and longevity. Evolutionary Anthropology: Issues News Reviews 9(4):156–185. 10.1002/1520-6505(2000)9:4%253C156::AID-EVAN5%253E3.0.CO;2-7

[CR97] Kappelman J (1996) The evolution of body mass and relative brain size in fossil hominids. J Hum Evol 30(3):243–276. 10.1006/jhev.1996.0021

[CR98] Keyser AW, Menter CG, Moggi-Cecchi J, Rayne Pickering P, Berger LR (2000) Drimolen: A new hominid-bearing site in Gauteng, South Africa. South Afr J Sci 96(4):193–197. 10.10520/AJA00382353_8903

[CR99] Kochiyama T, Ogihara N, Tanabe HC, Kondo O, Amano H, Hasegawa K, Akazawa T (2018) Reconstructing the Neanderthal brain using computational anatomy. Sci Rep 8(1):6296. 10.1038/s41598-018-24331-0 29700382 10.1038/s41598-018-24331-0 PMC5919901

[CR101] Kubo D, Tanabe HC, Kondo O, Ogihara N, Yogi A, Murayama S, Ishida H (2014) Cerebellar Size Estimation from Endocranial Measurements: An Evaluation Based on MRI Data. In: Akazawa T, Ogihara N, Tanabe HC, Terashima H (eds) Dynamics of Learning in Neanderthals and Modern Humans Volume 2: Cognitive and Physical Perspectives. Springer Japan, Tokyo, pp 209–215. 10.1007/978-4-431-54553-8_24

[CR100] Kubo D, T Kono R, Kaifu Y (2013) Brain size of Homo floresiensis and its evolutionary implications. Proc Royal Soc B: Biol Sci 280(1760):20130338. 10.1098/rspb.2013.0338 10.1098/rspb.2013.0338 PMC3652458 23595271

[CR102] Kuzawa CW, Chugani HT, Grossman LI, Lipovich L, Muzik O, Hof PR, Lange N (2014) Metabolic costs and evolutionary implications of human brain development. *Proceedings of the National Academy of Sciences*, *111*(36), 13010–13015. 10.1073/pnas.1323099111 10.1073/pnas.1323099111 PMC4246958 25157149

[CR103] Labra N, Mounier A, Leprince Y, Rivière D, Didier M, Bardinet E, Balzeau A (2024) What do brain endocasts tell us? A comparative analysis of the accuracy of sulcal identification by experts and perspectives in palaeoanthropology. J Anat 244(2):274–296. 10.1111/joa.13966 37935387 10.1111/joa.13966 PMC10780157

[CR104] Laland KN, Hoppitt W (2003) Do animals have culture? Evol Anthropol 12(3):150–159

[CR105] Leigh SR (1994) Relations between captive and noncaptive weights in anthropoid primates. Zoo Biol 13(1):21–43. 10.1002/zoo.1430130105

[CR106] Leigh SR (2006) Brain ontogeny and life history in *Homo erectus*. J Hum Evol 50(1):104–108. 10.1016/j.jhevol.2005.02.008 16226296 10.1016/j.jhevol.2005.02.008

[CR107] Lordkipanidze D, Ponce de León MS, Margvelashvili A, Rak Y, Rightmire GP, Vekua A, Zollikofer CPE (2013) A Complete Skull from Dmanisi, Georgia, and the Evolutionary Biology of Early Homo. Science 342(6156):326–331. 10.1126/science.1238484 24136960 10.1126/science.1238484

[CR108] MacLean EL, Hare B, Nunn CL, Addessi E, Amici F, Anderson RC, Zhao Y (2014) The evolution of self-control. *Proceedings of the National Academy of Sciences*, *111*(20), E2140–E2148. 10.1073/pnas.1323533111 10.1073/pnas.1323533111 PMC4034204 24753565

[CR109] Mahoney P, McFarlane G, Taurozzi AJ, Madupe PP, O’Hara MC, Molopyane K, Berger L (2024) Human-like enamel growth in *Homo naledi*. Am J Biol Anthropol 184(1):e24893. 10.1002/ajpa.24893 38180115 10.1002/ajpa.24893

[CR110] Marchi D, Walker CS, Wei P, Holliday TW, Churchill SE, Berger LR, DeSilva JM (2017) The thigh and leg of *Homo naledi*. J Hum Evol 104:174–204. 10.1016/j.jhevol.2016.09.005 27855981 10.1016/j.jhevol.2016.09.005

[CR111] Martinón-Torres M, Garate D, Herries AIR, Petraglia MD (2023) No scientific evidence that *Homo naledi* buried their dead and produced rock art. J Hum Evol 103464. 10.1016/j.jhevol.2023.103464 10.1016/j.jhevol.2023.103464 37953122

[CR112] Mcbrearty S, Brooks AS (2000) The revolution that wasn’t: A new interpretation of the origin of modern human behavior. J Hum Evol 39(5):453–563. 10.1006/jhev.2000.0435 11102266 10.1006/jhev.2000.0435

[CR113] McFarlin SC, Barks SK, Tocheri MW, Massey JS, Eriksen AB, Fawcett KA, Sherwood CC (2013) Early Brain Growth Cessation in Wild Virunga Mountain Gorillas (*Gorilla beringei beringei*). Am J Primatol 75(5):450–463. 10.1002/ajp.22100 23208801 10.1002/ajp.22100

[CR114] McHenry HM (1975) Fossil hominid body weight and brain size. Nature 254(5502):686–688. 10.1038/254686a0 804665 10.1038/254686a0

[CR115] McHenry HM (1976) Early hominid body weight and encephalization. Am J Phys Anthropol 45(1):77–83. 10.1002/ajpa.1330450110

[CR116] McHenry HM, Coffing K (2000) *Australopithecus* to *Homo*: Transformations in Body and Mind. *Annual Review of Anthropology*, *29*(Volume 29, 2000), 125–146. 10.1146/annurev.anthro.29.1.125

[CR118] Mingazzini G (1928) Beitrag zur Morphologie der äußeren Großhirnhemisphärenoberfläche bei den Anthropoiden (Schimpanse und Orang). Arch Psychiatr Nervenkrankh 85(1):1–219. 10.1007/BF01814392

[CR117] Mitteroecker P, Gunz P, Windhager S, Schaefer K (2013) A brief review of shape, form, and allometry in geometric morphometrics, with applications to human facial morphology. Hystrix Italian J Mammalogy 24(1):59–66. 10.4404/hystrix-24.1-6369

[CR119] Moss ML, Young RW (1960) A functional approach to craniology. Am J Phys Anthropol 18(4):281–292. 10.1002/ajpa.1330180406 13773136 10.1002/ajpa.1330180406

[CR120] Neubauer S (2014) Endocasts: Possibilities and Limitations for the Interpretation of Human Brain Evolution. Brain Behav Evol 84(2):117–134. 10.1159/000365276 25247826 10.1159/000365276

[CR121] Neubauer S, Gunz P (2018) Endocasts and the Evo-Devo Approach to Study Human Brain Evolution. In: Bruner E, Ogihara N, Tanabe HC (eds) Digital Endocasts: From Skulls to Brains. Springer Japan, Tokyo, pp 173–190. 10.1007/978-4-431-56582-6_12

[CR122] Neubauer S, Gunz P, Hublin J-J (2010) Endocranial shape changes during growth in chimpanzees and humans: A morphometric analysis of unique and shared aspects. J Hum Evol 59(5):555–566. 10.1016/j.jhevol.2010.06.011 20727571 10.1016/j.jhevol.2010.06.011

[CR123] Neubauer S, Gunz P, Leakey L, Leakey M, Hublin J-J, Spoor F (2018a) Reconstruction, endocranial form and taxonomic affinity of the early *Homo* calvaria KNM-ER 42700. J Hum Evol 121:25–39. 10.1016/j.jhevol.2018.04.005 29706231 10.1016/j.jhevol.2018.04.005

[CR124] Neubauer S, Gunz P, Weber GW, Hublin J-J (2012) Endocranial volume of *Australopithecus africanus*: New CT-based estimates and the effects of missing data and small sample size. J Hum Evol 62(4):498–510. 10.1016/j.jhevol.2012.01.005 22365336 10.1016/j.jhevol.2012.01.005

[CR125] Neubauer S, Hublin J-J, Gunz P (2018b) The evolution of modern human brain shape. Sci Adv 4(1):eaao5961. 10.1126/sciadv.aao5961 29376123 10.1126/sciadv.aao5961 PMC5783678

[CR126] Pang JC, Aquino KM, Oldehinkel M, Robinson PA, Fulcher BD, Breakspear M, Fornito A (2023) Geometric constraints on human brain function. Nature 618(7965):566–574. 10.1038/s41586-023-06098-1 37258669 10.1038/s41586-023-06098-1 PMC10266981

[CR127] Pereira-Pedro AS, Masters M, Bruner E (2017) Shape analysis of spatial relationships between orbito-ocular and endocranial structures in modern humans and fossil hominids. J Anat 231(6):947–960. 10.1111/joa.12693 29027198 10.1111/joa.12693 PMC5696126

[CR128] Pettitt P (2018) Hominin evolutionary thanatology from the mortuary to funerary realm: The palaeoanthropological bridge between chemistry and culture. Philosophical Trans Royal Soc B: Biol Sci 373(1754):20180212. 10.1098/rstb.2018.0212 10.1098/rstb.2018.0212 PMC6053978 30012742

[CR129] Pettitt P (2022) Did *Homo naledi* dispose of their dead in the Rising Star Cave system? South Afr J Sci 118(11/12). 10.17159/sajs.2022/15140

[CR130] Pettitt P (2024) The Cultural Ecology of Fear: Human Funerary Cognition in Evolutionary Perspective. In: Wynn T, Overmann KA, Coolidge FL (eds) Oxford Handbook of Cognitive Archaeology. Oxford University Press, p 0. 10.1093/oxfordhb/9780192895950.013.25

[CR131] Pickering R, Herries AIR, Woodhead JD, Hellstrom JC, Green HE, Paul B, Hancox PJ (2019) U–Pb-dated flowstones restrict South African early hominin record to dry climate phases. Nature 565(7738):226–229. 10.1038/s41586-018-0711-0 30464348 10.1038/s41586-018-0711-0

[CR132] Plate T, Heiberger R (2024) *abind: Combine Multidimensional Arrays*. Retrieved from https://cran.r-project.org/web/packages/abind/index.html

[CR133] Ponce de León MS, Bienvenu T, Marom A, Engel S, Tafforeau P, Warren A, Zollikofer JL, C. P. E (2021) The primitive brain of early *Homo*. Science 372(6538):165–171. 10.1126/science.aaz0032 33833119 10.1126/science.aaz0032

[CR134] Profico A, Buzi C, Melchionna M, Veneziano A, Raia P (2020) Endomaker, a new algorithm for fully automatic extraction of cranial endocasts and the calculation of their volumes. Am J Phys Anthropol 172(3):511–515. 10.1002/ajpa.24043 32187657 10.1002/ajpa.24043

[CR136] Randolph-Quinney PS (2015) A new star rising: Biology and mortuary behaviour of *Homo naledi*. South Afr J Sci 111(9/10):4–4. 10.17159/sajs.2015/a0122

[CR135] R Core Team (2021) R: A Language and Environment for Statistical Computing. R Foundation for Statistical Computing. Retrieved from https://cran.r-project.org/

[CR137] Rilling JK (2006) Human and nonhuman primate brains: Are they allometrically scaled versions of the same design? Evolutionary Anthropology: Issues News Reviews 15(2):65–77. 10.1002/evan.20095

[CR138] Rilling JK, Insel TR (1998) Evolution of the Cerebellum in Primates: Differences in Relative Volume among Monkeys, Apes and Humans. Brain Behav Evol 52(6):308–314. 10.1159/000006575 9807015 10.1159/000006575

[CR139] Robbins JL, Dirks PHGM, Roberts EM, Kramers JD, Makhubela TV, Hilbert-Wolf HL, Berger LR (2021) Providing context to the *Homo naledi* fossils: Constraints from flowstones on the age of sediment deposits in Rising Star Cave, South Africa. Chem Geol 567:120108. 10.1016/j.chemgeo.2021.120108

[CR140] Roth G, Dicke U (2005) Evolution of the brain and intelligence. Trends Cogn Sci 9(5):250–257. 10.1016/j.tics.2005.03.005 15866152 10.1016/j.tics.2005.03.005

[CR141] Ruff CB, Burgess ML, Squyres N, Junno J-A, Trinkaus E (2018) Lower limb articular scaling and body mass estimation in Pliocene and Pleistocene hominins. J Hum Evol 115:85–111. 10.1016/j.jhevol.2017.10.014 29331230 10.1016/j.jhevol.2017.10.014

[CR142] Ruff CB, Squyres N, Junno J-A (2020) Body mass estimation in hominins from humeral articular dimensions. Am J Phys Anthropol 173(3):480–499. 10.1002/ajpa.24090 32529636 10.1002/ajpa.24090

[CR143] Ruff CB, Wood BA (2023) The estimation and evolution of hominin body mass. Evolutionary Anthropology: Issues News Reviews 32(4):223–237. 10.1002/evan.21988 10.1002/evan.21988 37335778

[CR144] Scerri EML, Will M (2023) The revolution that still isn’t: The origins of behavioral complexity in *Homo sapiens*. J Hum Evol 179:103358. 10.1016/j.jhevol.2023.103358 37058868 10.1016/j.jhevol.2023.103358

[CR145] Schenker NM, Hopkins WD, Spocter MA, Garrison AR, Stimpson CD, Erwin JM, Sherwood CC (2010) Broca’s Area Homologue in Chimpanzees (*Pan troglodytes*): Probabilistic Mapping, Asymmetry, and Comparison to Humans. Cereb Cortex 20(3):730–742. 10.1093/cercor/bhp138 19620620 10.1093/cercor/bhp138 PMC2820707

[CR146] Schlager S (2017) Chapter 9 - Morpho and Rvcg – Shape Analysis in R: R-Packages for Geometric Morphometrics, Shape Analysis and Surface Manipulations. In G. Zheng, S. Li, & G. Székely (Eds.), *Statistical Shape and Deformation Analysis* (pp. 217–256). Academic Press. 10.1016/B978-0-12-810493-4.00011-0

[CR147] Schroeder L, Scott JE, Garvin HM, Laird MF, Dembo M, Radovčić D, Ackermann RR (2017) Skull diversity in the *Homo* lineage and the relative position of *Homo naledi*. J Hum Evol 104:124–135. 10.1016/j.jhevol.2016.09.014 27836166 10.1016/j.jhevol.2016.09.014

[CR148] Schubiger MN, Fichtel C, Burkart JM (2020) Validity of Cognitive Tests for Non-human Animals: Pitfalls and Prospects. *Frontiers in Psychology*, *11*. 10.3389/fpsyg.2020.01835 10.3389/fpsyg.2020.01835 PMC7488350 32982822

[CR149] Schwartz E, Nenning K-H, Heuer K, Jeffery N, Bertrand OC, Toro R, Langs G (2023) Evolution of cortical geometry and its link to function, behaviour and ecology. Nat Commun 14(1):2252. 10.1038/s41467-023-37574-x 37080952 10.1038/s41467-023-37574-x PMC10119184

[CR150] Scott N, Neubauer S, Hublin J-J, Gunz P (2014) A Shared Pattern of Postnatal Endocranial Development in Extant Hominoids. Evol Biol 41(4):572–594. 10.1007/s11692-014-9290-7

[CR151] Semaw S, Rogers MJ, Simpson SW, Levin NE, Quade J, Dunbar N, Everett M (2020) Co-occurrence of Acheulian and Oldowan artifacts with *Homo erectus* cranial fossils from Gona, Afar, Ethiopia. Sci Adv 6(10):eaaw4694. 10.1126/sciadv.aaw4694 32181331 10.1126/sciadv.aaw4694 PMC7056306

[CR153] Sherwood CC, Bauernfeind AL, Bianchi S, Raghanti MA, Hof PR (2012) Human brain evolution writ large and small. In *Progress in Brain Research*. Elsevier 195:237–254. 10.1016/B978-0-444-53860-4.00011-8 10.1016/B978-0-444-53860-4.00011-8 22230630

[CR154] Sherwood CC, Broadfield DC, Holloway RL, Gannon PJ, Hof PR (2003) Variability of Broca’s area homologue in African great apes: Implications for language evolution. Anat Record Part A: Discoveries Mol Cell Evolutionary Biology 271A(2):276–285. 10.1002/ar.a.10046 10.1002/ar.a.10046 12629670

[CR155] Sherwood CC, Subiaul F, Zawidzki TW (2008) A natural history of the human mind: Tracing evolutionary changes in brain and cognition. J Anat 212(4):426–454. 10.1111/j.1469-7580.2008.00868.x 18380864 10.1111/j.1469-7580.2008.00868.x PMC2409100

[CR156] Smaers JB, Rothman RS, Hudson DR, Balanoff AM, Beatty B, Dechmann DKN, Safi K (2021) The evolution of mammalian brain size. Sci Adv 7(18):eabe2101. 10.1126/sciadv.abe2101 33910907 10.1126/sciadv.abe2101 PMC8081360

[CR157] Smith BH (1986) Dental development in *Australopithecus* and early *Homo*. Nature 323(6086):327–330. 10.1038/323327a0

[CR158] Smith RJ, Jungers WL (1997) Body mass in comparative primatology. J Hum Evol 32(6):523–559. 10.1006/jhev.1996.0122 9210017 10.1006/jhev.1996.0122

[CR159] Smith TM (2013) Teeth and Human Life-History Evolution. *Annual Review of Anthropology*, *42*(Volume 42, 2013), 191–208. 10.1146/annurev-anthro-092412-155550

[CR160] Solhtalab A, Foroughi AH, Pierotich L, Razavi MJ (2025) Stress landscape of folding brain serves as a map for axonal pathfinding. Nat Commun 16(1):1187. 10.1038/s41467-025-56362-3 39885152 10.1038/s41467-025-56362-3 PMC11782574

[CR161] Stout D, Chaminade T (2012) Stone tools, language and the brain in human evolution. Philosophical Trans Royal Soc B: Biol Sci 367(1585):75–87. 10.1098/rstb.2011.0099 10.1098/rstb.2011.0099 PMC3223784 22106428

[CR162] Stout D, Hecht EE (2017) Evolutionary neuroscience of cumulative culture. *Proceedings of the National Academy of Sciences*, *114*(30), 7861–7868. 10.1073/pnas.1620738114 10.1073/pnas.1620738114 PMC5544267 28739892

[CR163] Strick PL, Dum RP, Fiez JA (2009) Cerebellum and Nonmotor Function. Annu Rev Neurosci 32(32, 2009):413–434. 10.1146/annurev.neuro.31.060407.125606 19555291 10.1146/annurev.neuro.31.060407.125606

[CR164] Striedter GF (2006) Précis of Principles of Brain Evolution. Behav Brain Sci 29(1):1–12. 10.1017/S0140525X06009010 16542524 10.1017/S0140525X06009010

[CR165] Syeda SM, Dunmore CJ, Skinner MM, Berger LR, Churchill SE, Zipfel B, Kivell TL (2025) Phalangeal cortical bone distribution reveals different dexterous and climbing behaviors in *Australopithecus sediba* and *Homo naledi*. Sci Adv 11(20):eadt1201. 10.1126/sciadv.adt1201 40367176 10.1126/sciadv.adt1201 PMC12077519

[CR166] Tallinen T, Chung JY, Rousseau F, Girard N, Lefèvre J, Mahadevan L (2016) On the growth and form of cortical convolutions. Nat Phys 12(6):588–593. 10.1038/nphys3632

[CR167] Tanabe HC, Kubo D, Hasegawa K, Kochiyama T, Kondo O (2018) Cerebellum: Anatomy, Physiology, Function, and Evolution. In: Bruner E, Ogihara N, Tanabe HC (eds) Digital Endocasts: From Skulls to Brains. Springer Japan, Tokyo, pp 275–289. 10.1007/978-4-431-56582-6_18

[CR168] Toro R, Burnod Y (2005) A Morphogenetic Model for the Development of Cortical Convolutions. Cereb Cortex 15(12):1900–1913. 10.1093/cercor/bhi068 15758198 10.1093/cercor/bhi068

[CR169] Val A (2016) Deliberate body disposal by hominins in the Dinaledi Chamber, Cradle of Humankind, South Africa? J Hum Evol 96:145–148. 10.1016/j.jhevol.2016.02.004 27039664 10.1016/j.jhevol.2016.02.004

[CR170] Van Essen DC (1997) A tension-based theory of morphogenesis and compact wiring in the central nervous system. Nature 385(6614):313–318. 10.1038/385313a0 9002514 10.1038/385313a0

[CR171] Van Essen DC (2020) A 2020 view of tension-based cortical morphogenesis. *Proceedings of the National Academy of Sciences*, *117*(52), 32868–32879. 10.1073/pnas.2016830117 10.1073/pnas.2016830117 PMC7780001 33323481

[CR172] VanSickle C, Cofran Z, Hunt D (2020) Did Neandertals have large brains? Factors affecting endocranial volume comparisons. Am J Phys Anthropol 173(4):768–775. 10.1002/ajpa.24124 33459351 10.1002/ajpa.24124

[CR173] Walker AE, Fulton JF (1936) The External Configuration of the Cerebral Hemispheres of the Chimpanzee. J Anat 71(Pt 1):105–116 17104620 PMC1252789

[CR174] Walker CS, Cofran Z, Grabowski M, Marchi D, Cook RW, Churchill SE, DeSilva JM (2019) Morphology of the *Homo naledi* femora from Lesedi. Am J Phys Anthropol 170(1):5–23. 10.1002/ajpa.23877 31228254 10.1002/ajpa.23877

[CR175] Weaver A (2005) Reciprocal evolution of the cerebellum and neocortex in fossil humans. Proc Natl Acad Sci 102(10):3576–3580 15731345 10.1073/pnas.0500692102 PMC553338

[CR176] Weber GW (2015) Virtual Anthropology. Am J Phys Anthropol 156(S59):22–42. 10.1002/ajpa.22658 25418603 10.1002/ajpa.22658

[CR177] Weber GW, Bookstein FL (2011) *Virtual Anthropology*. New York: Springer. Retrieved from https://link.springer.com/book/9783211486474

[CR178] Wynn T, Coolidge FL (2016) Archeological insights into hominin cognitive evolution. Evolutionary Anthropology: Issues News Reviews 25(4):200–213. 10.1002/evan.21496 10.1002/evan.21496 27519459

[CR179] Yin S, Liu C, Choi G, Jung Y, Heuer K, Toro R, Mahadevan L (2025) Morphogenesis and morphometry of brain folding patterns across species. eLife 14. 10.7554/eLife.107138.2 10.7554/eLife.107138 PMC12747518 41459643

[CR180] Zhang K, Sejnowski TJ (2000) A universal scaling law between gray matter and white matter of cerebral cortex. Proc Natl Acad Sci 97(10):5621–5626. 10.1073/pnas.090504197 10792049 10.1073/pnas.090504197 PMC25878

[CR181] Zollikofer CPE, Beyrand V, Lordkipanidze D, Tafforeau P, Ponce de León MS (2024) Dental evidence for extended growth in early *Homo* from Dmanisi. Nature 635(8040):906–911. 10.1038/s41586-024-08205-2 39537931 10.1038/s41586-024-08205-2 PMC11602720

[CR183] Zollikofer CPE, Bienvenu T, Beyene Y, Suwa G, Asfaw B, White TD, Ponce de León MS (2022) Endocranial ontogeny and evolution in early Homo sapiens: The evidence from Herto, Ethiopia. Proceedings of the National Academy of Sciences, 119(32), e2123553119. 10.1073/pnas.2123553119 10.1073/pnas.2123553119 PMC9371682 35914174

[CR184] Zollikofer CPE, Bienvenu T, Ponce de León MS (2017) Effects of cranial integration on hominid endocranial shape. J Anat 230(1):85–105. 10.1111/joa.12531 27503252 10.1111/joa.12531 PMC5192801

[CR182] Zollikofer CPE, Ponce de León MS (2013) Pandora’s growing box: Inferring the evolution and development of hominin brains from endocasts. Evolutionary Anthropology: Issues News Reviews 22(1):20–33. 10.1002/evan.21333 10.1002/evan.21333 23436646

