# Supplementary material for: Brain structure and function in Homo naledi
Source: Brain Struct Funct. 2026 Jun 15;231(6):89. doi: 10.1007/s00429-026-03129-1 (PMC13269317; doi:10.1007/s00429-026-03129-1)
Supplement: Supplementary file 1 — Supplementary Material 1 [file 429_2026_3129_MOESM1_ESM.docx]

**Supporting Information**

For

**Brain Structure and Function in *Homo naledi***

Zachary Cofran, Shawn Hurst, John Hawks

**Figure S1**

Hierarchical clustering analysis (UPGMA) including the entire adult modern human sample. All humans fall within the same cluster. The topological differences between this full-sample tree and that using only the individual closest to the human average are: 1) with the full sample, humans fall outside a fossil *Homo* cluster, whereas in the reduced sample KNM-ER 1813 is the outgroup to the rest of *Homo*; 2) with the full sample, KNM-ER 1470 is in a cluster including LES1 and Indonesian *H. erectus*, whereas in the reduced sample KNM-ER 1470 and the average human form an outgroup to a cluster including LES1 and all *H. erectus*. In either case, LES1 falls within the Indonesian *H. erectus* cluster. Colors correspond to taxa as in previous figures.

**Figure S2**

Principal components analysis of endocast shape among humans and the full fossil sample including non-adult specimens Mojokerto and KNM-ER 42700. Triangle meshes illustrate the negative (white) and positive (gray) extremes of shape variation along the first two principal component (PC) axes. PC1 distinguishes modern humans from the fossil sample, especially adult *H. erectus*. PC2 is driven largely by intraspecific variation. The high PC1-2 scores of Mojokerto and KNM-ER 42700 compared to other *H. erectus* likely illustrate ontogenetic variation in that taxon.
